# Exercise in atherosclerosis: its beneficial effects and underlying mechanism

**DOI:** 10.3389/fcell.2025.1598794

**Published:** 2025-08-11

**Authors:** Meng-ting Yin, Liang Guo

**Affiliations:** ^1^ Key Laboratory of Exercise and Health Sciences of the Ministry of Education, Shanghai University of Sport, Shanghai, China; ^2^ School of Exercise and Health and Collaborative Innovation Center for Sports and Public Health, Shanghai University of Sport, Shanghai, China; ^3^ Shanghai Key Lab of Human Performance, Shanghai University of Sport, Shanghai, China; ^4^ Shanghai Frontiers Science Research Base of Exercise and Metabolic Health, Shanghai University of Sport, Shanghai, China

**Keywords:** exercise, atherosclerosis, exerkines, browning of adipose tissue, cell death, microRNAs, immune function

## Abstract

Atherosclerosis represents a complex interplay of inflammatory and metabolic processes, in which oxidative stress, endothelial inflammation, the phenotypic transition of smooth muscle cells (SMCs), and the conversion of macrophages into foam cells are involved. In contrast to pharmacological interventions, exercise emerges as a viable, cost-effective, and low-risk strategy to alleviate the progression of atherosclerosis. Exercise exerts beneficial effects on atherosclerosis through modulation of diverse pathways, including exerkines, browning of adipose tissue, the renin-angiotensin system (RAS), metabolites, gut microbiota, cell death pathways, microRNAs, nervous system, and immune function. The beneficial impacts of exercise on atherosclerosis and the mechanisms behind them will be examined here. Fully understanding the effects and mechanisms of exercise in reducing atherosclerosis might open doors to developing safe and effective interventions.

## 1 Introduction

For globally, atherosclerosis is a major cause of illness and death, defined by the ongoing accumulation of lipids and fibrous tissue in arterial walls. Atherosclerosis is a global health issue affecting individuals in the age range of 30–79 years, with an estimated global prevalence of 27.6% in 2020. Males have a higher prevalence than females, and it rises with age (Song et al., 2020a). According to 2020 global data, approximately 27.6% (1.067 billion) of individuals aged 30–79 years exhibited carotid intima-media thickening (≥1.0 mm), with 21.1% (816 million) presenting carotid plaques and 1.5% (57.79 million) showing stenosis (≥50%). Notably, the number of cases has increased by more than 57% since 2000 (Song et al., 2020b). Hence, elucidating the pathogenesis of atherosclerosis and developing early intervention strategies to alleviate its progression have become imperative. Atherosclerosis is a multifaceted pathological process that progresses through several stages, beginning with the retention and oxidation of low-density lipoprotein (LDL) within the arterial intima, which triggers a localized inflammatory response. Oxidized low-density lipoprotein (ox-LDL) activates monocytes, prompting their migration into the intima, where they differentiate into macrophages. These macrophages engulf ox-LDL, transforming into foam cells that release pro-inflammatory cytokines, thereby exacerbating local inflammation. Concurrently, vascular smooth muscle cells (VSMCs) migrate from the medial layer to the intima, proliferate, and synthesize collagen to form a fibrous cap in an attempt to stabilize the plaque. However, as inflammation persists and cellular apoptosis progresses, the fibrous cap gradually weakens, rendering the plaque unstable and prone to rupture. Ultimately, plaque rupture can lead to thrombus formation, resulting in severe cardiovascular events such as myocardial infarction and stroke (Libby, 2021a; Ajoolabady et al., 2024). Overall, the pathophysiology of atherosclerosis includes the oxidation of LDL, inflammation of endothelial cells (ECs), phenotypic transformation of VSMCs, and macrophages turning into foam cells (Libby, 2021b). Risk factors for atherosclerosis include poor dietary habits, high LDL cholesterol, hypertension, smoking, disrupted sleep patterns, sedentary lifestyles, and gut microbiota dysbiosis (Libby, 2021b). Early action to tackle these risk factors may decrease the rate of atherosclerosis in adults. Statins act as competitive inhibitors of HMG-CoA reductase, the enzyme that limits the rate of cholesterol production in hepatocytes, thereby decreasing cholesterol synthesis. This decrease subsequently activates a feedback loop that boosts the quantity and function of LDL receptors on the cell surface, resulting in improving clearance of serum cholesterol and a reduction in its level (Kim et al., 2024). Additionally, proprotein convertase subtilisin/kexin type 9 (PCSK9) is a key regulator of low-density lipoprotein receptor (LDLR) activity, increasing circulating LDL cholesterol (LDL-C) levels by regulating LDLR degradation (Barbieri et al., 2024). Long-term use of PCSK9 inhibitors in combination with statins exhibits a synergistic effect that further reduces the risk of cardiovascular events (O’Donoghue et al., 2022). PCSK9 inhibitors primarily include monoclonal antibodies (e.g., alirocumab and evolocumab) and small interfering RNA (e.g., inclisiran). The monoclonal antibodies bind to PCSK9 extracellularly, preventing its interaction with LDLR and thus inhibiting LDLR degradation. In contrast, small interfering RNA suppresses hepatic synthesis of PCSK9 via RNA interference, reducing both intracellular and extracellular PCSK9 levels and indirectly enhancing LDLR expression. Ultimately, these agents facilitate LDL-C clearance, stabilize atherosclerotic plaques, reduce inflammation, and promote plaque regression (Corsini et al., 2025). Overall, atherosclerosis remains a major global health burden, necessitating the exploration of effective therapeutic approaches.

Exercise is a physical activity that is planned, structured, and repetitive, which can improve or maintain one or more components of physical fitness, contribute to mental wellbeing, and aid in the prevention and treatment of numerous diseases. Growing lines of evidence have demonstrate the beneficial effect of exercise on the amelioration of atherosclerosis. This review comprehensively examines the role and molecular mechanisms by which exercise alleviates atherosclerosis. The evidence demonstrates that exercise serves as a promising therapeutic strategy by mitigating inflammation, modulating immune responses, improving lipid metabolism, enhancing plaque stability, and restoring endothelial function in models with atherosclerosis. These synergistic effects collectively provide a multidimensional therapeutic approach to attenuate disease progression and reduce cardiovascular complications. Notably, different types, intensities, and durations of exercise exhibit distinct effects on the alleviation of atherosclerosis. Among these, long-term moderate-intensity aerobic exercises, such as running and swimming, are generally considered the most effective in improving lipid profiles and vascular function. Furthermore, when combined with healthy dietary patterns or lifestyle interventions like intermittent fasting, exercise may produce synergistic effects, offering a more comprehensive strategy for prevention and management of atherosclerosis.

## 2 Role and molecular mechanism of exercise-mediated alleviation of atherosclerosis

Exercise can alleviate atherosclerosis, in which multiple pathways and molecular mechanisms are invovled, demonstrating the significant effects of exercise in both human and animal models. Human studies have shown that aerobic exercise effectively regulates lipid metabolism by increasing high-density lipoprotein (HDL) while reducing LDL and triglycerides (TG). It also decreases carotid intima-media thickness (CIMT) and lowers inflammatory markers such as ​​c-reactive protein (CRP), thereby slowing atherosclerosis progression (Mendoza and Lavie, 2022; Meyer-Lindemann et al., 2023a). Clinical observations further indicate that high-intensity interval training and long-term moderate-intensity aerobic exercise significantly reduce coronary plaque volume and enhance plaque stability. Animal experiments have further validated the protective role of exercise (Wang et al., 2022a; Byrkjeland et al., 2016a). In ApoE^−/−^ mouse models, exercise stabilizes atherosclerotic plaques by reducing the lipid core, increasing collagen content, and thickening the fibrous cap, while also lowering matrix metalloproteinase (MMP) activity to inhibit plaque rupture risk (Vergallo et al., 2020). Additionally, 12 weeks of swimming exercise in ApoE^−/−^ mice resulted in attenuated weight gain, improved arterial structural integrity, and reduced atherosclerotic lesion burden, accompanied by decreased serum concentrations of total cholesterol (TC), TG, soluble intercellular adhesion molecule-1 (ICAM-1), matrix metalloproteinase-9 (MMP-9), and interleukin-6 (IL-6), collectively mitigating the progression of atherosclerosis (Li et al., 2020a).

In summary, exercise is well-established to alleviate atherosclerosis by modulating blood lipid profiles, reducing CIMT, and mitigating inflammation, thereby providing a robust foundation for non-pharmacological intervention.​ The alleviation of atherosclerosis through exercise involves an extremely complex molecular mechanism. This section will focus on elucidating the multiple mechanisms by which exercise mitigates atherosclerosis, including exercise-induced factors, browning of adipose tissue, the renin-angiotensin system (RAS), metabolites, gut microbiota, cell death pathways, microRNAs, nervous system, immune function, and so on.

### 2.1 Potential involvement of exerkines in exercise-mediated alleviation of atherosclerosis

**FIGURE 1:**
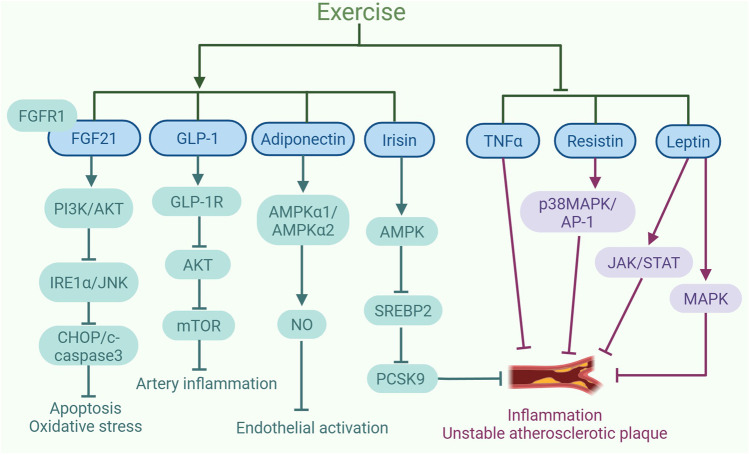
Exercise-induced exerkines alleviate the development of atherosclerosis. Exercise increases the expression and sensitivity of fibroblast growth factor 21 (FGF21). FGF21 binding to FGFR1 triggers the PI3K/AKT pathway, suppresses IRE1α/JNK activation, and lowers levels of apoptosis proteins like CHOP and cleaved-caspase3, mitigating oxidative stress and apoptosis. In addition, exercise leads to an increase in the levels of glucagon-like peptide-1 (GLP-1) secreted by intestinal L cells. The activation of GLP-1R by GLP-1 can inhibit AKT to reduce mTOR activity, which helps to control the inflammatory response in human coronary artery SMCs. Exercise upregulates the expression of adiponectin, which increases NO content by promoting AMPKα1/AMPKα2 expression, consequently inhibiting endothelial activation. Furthermore, exercise also increases the production of irisin, which can inhibit the expression of PCSK9 via AMPK/SREBP2 signaling pathway, leading to a reduction in inflammation and plaque instability. Moreover, exercise can decrease the expression of TNFα to inhibit inflammation and the instability of atherosclerotic plaques. Additionally, exercise can suppress the expression of resistin to inhibit the p38MAPK/AP-1 signaling pathway, thereby alleviating inflammation and the plaque instability associated with atherosclerosis. Exercise can also inhibit the expression of leptin, suppressing JAK/STAT and MAPK signaling pathways, thereby alleviating inflammation and plaque instability. The graph was created with biorender.com (agreement number: IE28KV91XQ).

Exercise can influence the levels of various exerkines, such as tumor necrosis factor α (TNFα), leptin, resistin, adiponectin, irisin, glucagon-like peptide-1 (GLP-1), fibroblast growth factor 21 (FGF21), among others. The alterations in these exerkines induced by exercise have been shown to mitigate atherosclerosis, as is summarized in Figure 1.

#### 2.1.1 TNFα

TNFα is known as a powerful pro-inflammatory cytokine that significantly contributes to the development of atherosclerosis (Rolski and Blyszczuk, 2020). TNFα is frequently employed to stimulate cellular inflammation in order to establish an inflammatory cell model of atherosclerosis. Human studies indicate that people with atherosclerosis exhibit higher serum levels of TNFα, and the concentration of TNFα in unstable atherosclerotic plaques is notably greater than in those with stable plaques. An investigation is performed to understand how TNFα functions at the molecular level in atherosclerosis (Zhang et al., 2014). The experiments performed *in vitro* demonstrated that TNFα stimulated the phagocytosis of LDL by human umbilical vein endothelial cells (HUVECs) and promoted the retention of LDL beneath the vascular wall, thus accelerating atherosclerosis progression. A study finds that ApoE^−/−^ mice injected with TNFα has five times higher plaque area than untreated ApoE^−/−^ mice. Endurance exercise intervention significantly increased the levels of short-chain fatty acids (SCFAs) in the aorta of mice with western diet (WD) -induced atherosclerosis, thereby reducing the production of aortic TNFα by inhibiting inflammatory signaling pathways. Additionally, exercise improved lipid metabolism by mitigating obesity, which contributed to decreased inflammatory responses and, consequently, lowered systemic TNFα levels (Huang et al., 2022). As a result, exercise reduces TNFα expression, which eases the progression of atherosclerosis.

#### 2.1.2 Leptin

Secreted by adipocytes, the hormone leptin is essential for energy regulation, inflammation control, vascular health, and the development of new blood vessels (Denver et al., 2011). In a comprehensive review, leptin is portrayed as a potential modulator of atherosclerosis, the impact of which is contingent upon its concentration in the blood (Raman and Khanal, 2021). Leptin controls energy balance and appetite at physiological levels, but in mice with obesity and diabetes, its elevated levels lead to hyperleptinemia, which supports atherosclerosis development. High levels of leptin activate the long form of leptin receptors (Ob-Rb) on ECs, SMCs, and macrophages, initiating signaling pathways like janus kinase/signal transducer and activator of transcription (JAK/STAT) and mitogen-activated protein kinase (MAPK), which contribute to inflammation and vascular damage. Therefore, the connection between leptin levels and atherosclerosis is complex, involving multiple cell types and molecular pathways, providing fresh insights for preventing and treating cardiovascular diseases (CVDs).

Interestingly, exercise can decrease leptin levels in atherosclerotic mice (Sousa et al., 2021a). In a study, a 6-week running regimen is implemented on ApoE^−/−^ mice, resulting in significant alleviation of atherosclerosis in the mouse model (Frodermann et al., 2019). This beneficial outcome is largely attributed to the decreased leptin levels in the bloodstream and tibia, alongside the inhibition of hyperlipidemia-associated leukocytosis progression, a decline in the accumulation of immune cells in the aorta, accompanied by smaller atherosclerotic plaques at the aortic root. Furthermore, a study demonstrates that aerobic exercise training reduces the protein levels of leptin in perivascular adipose tissue (PVAT) of obese mice, potentially enhancing the antioxidant response of PVAT and safeguarding vascular health (Sousa et al., 2021b). Consistent with findings from animal studies, research in humans has also demonstrated that exercise can reduce leptin expression, thereby alleviating atherosclerosis. A 12-week combined endurance and aerobic exercise intervention is conducted on obese adolescent girls in a human study. The results showed that plasma leptin levels significantly decreased after the exercise intervention. Additionally, the exercise significantly reduces arterial stiffness in obese adolescent girls, suggesting that the reduction in leptin levels is associated with improved arterial function, which may contribute to a reduced risk of atherosclerosis (Wong et al., 2018). Overall, investigations have revealed that engaging in exercise can diminish leptin levels in those with atherosclerosis, thereby reducing the pathological development of atherosclerosis.

#### 2.1.3 Resistin

Resistin, an adrenal protein, is released by diverse cellular sources and contributes to vascular inflammation, lipid accumulation, and plaque vulnerability. Resistin can act on ECs, VSMCs, and macrophages to exert pro-atherosclerotic effects, thereby contributing to cardiovascular damage such as dyslipidemia, atherosclerotic plaque rupture, and ventricular remodeling (Zhou et al., 2021). A recent human survey reveals elevated circulating resistin concentrations in patients with atherosclerosis (Cai et al., 2022). These molecular mechanisms collectively promote the development and destabilization of atherosclerotic plaques, revealing the multifaceted roles of resistin in the progression of atherosclerosis.

Exercise can mitigate the progression of atherosclerosis by reducing the level of resistin. In a study, Kadoglou et al. recruited 60 patients with type 2 diabetes who are overweight/obese (BMI>25 kg/m^2^) (Kadoglou et al., 2007). The participants are split into a control cohort and an exercise cohort, and the patients in the exercise group receives aerobic exercise training for 16 weeks. The experimental results show that the exercise group has significantly lower resistin levels than the control group. In addition, compared with ApoE^−/−^ mice that do not engage in exercise, the ApoE^−/−^ mice with 12 weeks of swimming exercise have significantly lower resistin levels in the aorta, less endothelial damage and reduced the severity of atherosclerosis (Cai et al., 2018). However, compared to other adipokines, the specific contribution of resistin to exercise-mediated protective role against atherosclerosis remains incompletely understood, and inter-individual variability in resistin response to exercise interventions adds further complexity to its study. Therefore, although resistin represents a promising target, additional mechanistic research is needed to clarify its role in exercise-mediated alleviation of atherosclerosis. Hence, exercise can decrease resistin expression, which may help mitigate the progression of atherosclerosis.

#### 2.1.4 Adiponectin

Adiponectin, one of the abundant hormones secreted by adipocytes, exhibits insulin sensitivity, anti-atherosclerotic and anti-inflammatory properties. Upregulation of adiponectin expression has been shown to confer protection against atherosclerotic plaque formation (Ahmadi et al., 2011). Numerous studies have indicated a relationship between exercise and elevated adiponectin levels. Exercise enhances the activation of intracellular signaling molecules, such as AMPKα1 and AMPKα2, within the adiponectin pathway. This boost in activity leads to greater availability of nitric oxide (NO), which in turn helps eliminate reactive oxygen species (ROS), decreases the levels of adhesion molecules, and mitigates EC activation. Adiponectin guards ECs against inflammation indirectly by curbing lipid buildup within macrophages, facilitating M2 macrophage polarization, and dampening toll-like receptor 4 (TLR4)-induced activation of ECs. Studies have shown that an 8-week aerobic exercise regimen enhances cardiac contractile function in WT mice. The specific molecular mechanisms likely involve adiponectin-mediated promotion of endothelial nitric oxide synthase (eNOS) activity, facilitation of VSMCs differentiation, suppression of vascular inflammation, reduction of cardiac cell apoptosis, and enhancement of cardiac and vascular function by activating the AMP-activated protein kinase (AMPK) pathway (Caldwell et al., 2021; Chen M. et al., 2023). Additionally, an 8-week sustained aerobic treadmill exercise regimen effectively upregulates adiponectin receptor (AdipoR1) protein expression and activates eNOS via the AMPK pathway. This activation enhances NO production and/or bioavailability while concurrently reducing ROS formation. The cumulative outcome of these changes is the restoration of vascular reactivity, a decrease in inflammatory and oxidative stress responses, and a significant counteraction against the development of atherosclerosis. Therefore, exercise can elevate adiponectin levels, which helps alleviate the progression of atherosclerosis.

#### 2.1.5 Irisin

Irisin is a myokine that is produced in response to physical exercise. Exercise can enhance the expression of peroxisome proliferator-activated receptor gamma coactivator 1-alpha (PGC-1α) in skeletal muscle, which in turn triggers the breakdown of fibronectin type III domain-containing protein 5 (FNDC5). Ultimately, this process results in irisin production (Chae et al., 2024). In cell experiments of ox-LDL-induced vascular ECs inflammation, irisin has the ability to activate the AMPK signaling cascade, suppress NF-κB p65 phosphorylation and inflammation-related gene expression, and exhibit a protective effect on vascular function. Furthermore, irisin suppresses PCSK9 expression via AMPK pathway activation, thereby reducing the activity of sterol regulatory element-binding protein 2 (SREBP2). This mechanism contributes to the alleviation of vascular EC inflammation induced by ox-LDL (Wang et al., 2023a). Elevated endogenous levels of irisin have been demonstrated to decrease arterial stiffness in both humans and animals by boosting the phosphorylation of AMPK, AKT, and eNOS within the arteries, along with increasing cycling levels of nitrite/nitrate (Inoue et al., 2020). Another study found that 6 weeks of moderate-intensity aerobic exercise significantly improved atherosclerotic risk markers in severely obese patients, including reductions in TC, LDL-C, and ox-LDL, as well as increased high-density lipoprotein cholesterol (HDL-C) levels, alongside decreased inflammatory marker CRP and enhanced antioxidant enzyme paraoxonase-1 (PON1) activity, whereas low-intensity exercise only partially improved the above metabolic parameters. Moderate-intensity exercise also specifically elevated irisin levels, which has potential anti-atherosclerotic effects, and reduced the pro-inflammatory factor lipocalin-2 (LCN2). Combined with improvements in cardiorespiratory fitness and exercise tolerance, these changes collectively played a multitarget vascular protective role (Horváth et al., 2024). Thus, exercise has the potential to elevate irisin levels to ameliorate atherosclerosis.

#### 2.1.6 GLP-1

GLP-1, secreted by intestinal L-cells as a peptide hormone, exerts various metabolic effects. The increase in active GLP-1 levels helps to improve glycemic control and insulin secretion (Ellingsgaard et al., 2020). A human study reveals that by examining the proportions of total macrophages (CD14^+^), M1 macrophages (CD14^+^CD80^+^), and M2 macrophages (CD14^+^CD206+), as well as the differences in surface GLP-1 receptor (GLP-1R) expression in peripheral blood between coronary heart disease patients and healthy control individuals, and found that GLP-1 promotes M2 macrophage polarization and alleviates atherosclerosis through its interaction with the GLP-1R (Yang L. et al., 2021). Additionally, treatment with GLP-1R agonists (GLP-1RAs) has been shown to reduce ROS production, enhance mitochondrial function, decrease leukocyte-endothelial interactions and inflammation, and also reduce CIMT in people with type 2 diabetes (Luna-Marco et al., 2023). Furthermore, the GLP-1R agonist liraglutide (GlpNP) specifically targets CD11b+/CD11c+ cells in the circulation of ApoE^−/−^ mice and SMCs in aortic plaques, leading to reductions in plasma TG-rich lipoproteins, plaque burden, and plaque cholesterol (Maiseyeu et al., 2022). Under TNFα-induced inflammatory conditions, GLP-1RAs (such as Exendin-4 and GLP-1) suppress the production of MMPs in human coronary artery smooth muscle cells (hCASMCs) by inhibiting AKT-Thr308 phosphorylation. Additionally, they attenuate mammalian target of rapamycin (mTOR) activity through the inhibition of AKT-mediated prolin-rich akt substrate (PRAS40) phosphorylation, indicating that GLP-1R agonists (GLP-1RAs) help to control inflammation and immune responses in hCASMCs, improve vascular health, and may positively impact the treatment of atherosclerosis by modulating the AKT and mTOR signaling pathways (Gallego-Colon et al., 2018; Li BY. et al., 2022). These mechanisms suggest that GLP-1R agonists help regulate inflammatory and immune responses in vascular smooth muscle cells and may ameliorate the progression of atherosclerosis by modulating the AKT and mTOR signaling pathways.

Numerous studies have demonstrated that exercise elevates circulating levels of GLP-1 (Yan and Guo, 2023). For example, high-intensity cycling training significantly increases plasma GLP-1 concentrations in obese individuals, thereby contributing to weight loss (Q et al., 1985). In addition, acute swimming and short-term endurance training have been shown to upregulate serum GLP-1 secretion in healthy mice, leading to enhanced exercise performance (Wu et al., 2022). These findings suggest that elevated GLP-1 may contribute to exercise-mediated mitigation of atherosclerosis. However, current animal and human studies investigating the role of enhanced GLP-1 signaling in the alleviation of atherosclerosis by exercise remain limited and require further validation. In addition, although GLP-1-mediated weight loss is beneficial for metabolic health, its potential adverse effects, such as the reduction of muscle mass, should not be overlooked. Future research should aim to identify optimized exercise and nutritional strategies that maximize the benefits of GLP-1 while minimizing potential metabolic side effects (Conte et al., 2024).

#### 2.1.7 FGF21

FGF21 is a crucial modulator of energy metabolism, stimulating brown adipose tissue (BAT) and promoting the browning of white adipose tissue (WAT) (Tucker et al., 2023). This enhances fatty acid uptake, reduces plasma cholesterol, particularly non-HDL-C, decreases atherosclerotic lesion area, and improves plaque stability, thus alleviating hypercholesterolemia and potentially mitigating atherosclerotic cardiovascular disease (ASCVD) severity.

Exercise can increase FGF21 levels or enhance its activity. Regular physical exercise elevates FGF21 levels to regulate the immune response in atherosclerosis, thereby reducing plaque formation. A 6-week aerobic exercise training program increases the expression of FGF21 in the hearts of myocardial infarction (MI) mice. FGF21, by binding to its receptor FGFR1, activates the downstream PI3K/AKT signaling pathway, inhibits the activation of the inositol-requiring enzyme 1α (IRE1α)/JNK pathway, and reduces the expression of apoptosis-related proteins such as C/EBP-homologous protein (CHOP) and claved-caspase3, thereby suppressing oxidative stress and apoptosis. Systemic knockout of FGF21 attenuates the effects of aerobic exercise on oxidative stress and cellular apoptosis in these mice (Bo et al., 2021). Another study analyzes FGF21 levels in ApoE^−/−^ mice serum during the early and late phases of exercise. The findings indicate that during the initial stages of exercise, serum FGF21 expression significantly increased, while in the later phase, the sensitivity to FGF21 may have been enhanced through increased expression of FGF21 downstream effectors such as adiponectin, which facilitates more effective action of FGF21 even if its serum concentration decreased (Li XH. et al., 2022). Collectively, these findings suggest that FGF21 may mediate some of the beneficial effects of exercise on atherosclerosis, particularly through the PI3K/AKT signaling pathway and anti-apoptotic mechanisms. However, most existing data are limited to murine models, with few translational studies in humans. Therefore, there is a pressing need for further research, especially well-designed clinical trials, to elucidate the mechanistic role of exercise-induced FGF21 in human atherosclerosis and to explore the long-term therapeutic potential of FGF21 induced by exercise in the prevention and treatment of atherosclerosis.

### 2.2 Effect of exercise-regulated browning of adipose tissue in amelioration of atherosclerosis

Exercise can activate BAT and induce the browning of adipose tissue to improve lipid metabolism, enhance energy expenditure, and attenuate inflammatory responses, which exert protective effects against atherosclerosis.

Activating BAT is essential for sustaining energy balance and metabolic homeostasis. Recent research has shown that BAT is also vital for preventing and treating atherosclerosis. Under cold exposure, both mice and humans exhibit increased activation of BAT and the browning of WAT. The activation of BAT is believed to mitigate the progression of atherosclerosis by increasing energy expenditure and improving lipid metabolism. The browning of WAT, which refers to the transformation of WAT towards a BAT-like phenotype, is also a potential strategy against atherosclerosis. This transformation is usually accompanied by a rise in the production of uncoupling protein 1 (UCP1), a mitochondrial protein that increases energy expenditure and generates heat by uncoupling the respiratory chain. A study has shown that the browning of WAT induced by β3-adrenergic agonists in mouse models reduces plasma TG, TC, and LDL, while simultaneously elevating HDL levels. This process also triggers the release of anti-atherosclerotic adipokines, including adiponectin and FGF21 (Roth et al., 2021). In addition to the browning of WAT, the development of brown adipocytes within PVAT has also been shown to exert a protective effect against atherosclerosis. One study demonstrated that bone morphogenetic protein 4 (BMP4) can induce the transdifferentiation of white adipocytes into beige adipocytes, thereby promoting the browning of PVAT and enhancing its thermogenic capacity and metabolic activity. This browning process has been confirmed to suppress vascular inflammation and attenuate atherosclerotic plaque formation in ApoE^−/−^ mice. Mechanistically, BMP4 overexpression upregulates the expression of brown fat marker genes such as UCP1 and PGC-1α, while significantly reducing the levels of pro-inflammatory cytokines including IL-1β, IL-6, monocyte chemoattractant protein 1 (MCP-1), and TNFα. In contrast, BMP4 deficiency leads to a phenotypic shift of PVAT toward white adipose characteristics, accompanied by increased macrophage infiltration, endothelial activation, and exacerbated atherosclerosis. These findings underscore the pivotal role of BMP4 in orchestrating anti-inflammatory responses through PVAT browning and highlight its potential as a therapeutic target for atherosclerosis (Mu et al., 2021). In summary, BAT activation and adipose tissue browning show potential in combating atherosclerosis. These effects may be achieved through mechanisms such as improving metabolic parameters, increasing energy expenditure, promoting the secretion of healthy adipokines, and reducing inflammation. Therefore, promoting BAT activity and the browning of adipose tissues could be a novel approach for the management and prevention of atherosclerosis.

In addition to cold exposure and other substances that can promote BAT activity and induce browning of adipose tissues, exercise can also promote BAT activity and adipose tissue browning. Four weeks of voluntary wheel-running exercise in mice can induce UCP1 expression in brown adipocytes, thereby activating BAT and increasing thermogenesis (Schonke and Gabriel, 2022). Additionally, both types of exercise - aerobic and resistance - significantly induce the browning of inguinal white adipose tissue and retroperitoneal white adipose tissue (rpWAT) in mice. Specifically, both aerobic training and resistance training lead to a notable rise in the expression of vascular endothelial growth factor (VEGF) and UCP1, enhancing vascularization of the fat tissue, boosting fatty acid oxidation, and increasing the expression of genes linked to the characteristics of brown and beige adipocyte phenotypes such as PGC-1α (Picoli et al., 2020). 8 weeks of treadmill training elevates UCP1 in the thoracic PVAT (tPVAT) of obese Zucker rats, promoting the browning of PVAT (DeVallance et al., 2019). Additionally, in Sprague-Dawley rats with PVAT dysfunction induced by a high-fat diet (HFD), exercise training promotes the transcriptional activation of BMP4 and its related signaling components in PVAT, such as p38/MAPK, PGC-1α, and Smad5, which increases the BMP4 protein content in PVAT and activates downstream signaling pathways, thereby facilitating the browning of PVAT. This indicates that exercise improves vascular function through the browning of PVAT and could potentially serve as a safeguard against atherosclerosis (Liu et al., 2024). A cross-sectional study recruited patients undergoing 18F-FDG positron emission tomography/computed tomography (PET/CT) scans and finds that an increase in habitual physical activity levels is associated with higher BAT activity. This suggests that exercise may increase energy expenditure by activating BAT, thereby combating obesity. Additionally, the study finds that the higher the body mass index (BMI), the lower the BAT activity (Dinas et al., 2014). In conclusion, exercise in mice may alleviate atherosclerosis by promoting the activation of BAT and the browning of WAT.

### 2.3 Effect of exercise-regulated renin-angiotensin system (RAS) in the amelioration of atherosclerosis

Exercise effectively modulates the RAS by inhibiting the classical angiotensin-converting enzyme (ACE)/angiotensin II (Ang II)/Ang II type 1 receptor (AT1R) pathway while activating the protective angiotensin-converting enzyme 2 (ACE2)/angiotensin-(1-7) [Ang 1-7]/Mas receptor axis. The above regulatory effects help attenuate the progression of atherosclerosis by suppressing inflammation and restoring vascular homeostasis. This section reviews recent advances and potential mechanisms underlying the exercise-mediated modulation of RAS in alleviating atherosclerosis.

The RAS consists of renin, ACE, and angiotensinogen (ATG). Renin initiates the hydrolysis of ATG to generate angiotensin I (Ang I), further transformed by ACE into Ang II, representing the classical branch of the RAS. Ang II is a potent vasoconstrictor that elevates blood pressure through binding to its receptors, particularly the AT1R. Angiotensin II not only drives atherosclerosis development but also exacerbates it through inflammation, cellular multiplication, and increased oxidative stress. Additionally, Ang II activates platelets and increases blood coagulability, further exacerbating the process of atherosclerosis. However, the RAS also includes a counterbalancing branch, known as the non-classical or protective branch, comprising ACE2 and its product Ang 1-7. ACE2 plays a crucial role in transforming Ang II into Ang 1-7. Ang 1-7, a vasodilatory peptide, combats atherosclerosis by inhibiting inflammation, oxidation, and proliferation via receptor Mas activation (Klersy et al., 2025). In a cross-sectional study, levels of serum RAS components are compared between female patients with rheumatoid arthritis (RA) and healthy females. The study reveals higher plasma Ang II, Ang 1-7, and ACE levels in RA patients compared to the control group. Furthermore, RA patients exhibit a higher ACE/ACE2 ratio and a lower Ang II/Ang 1-7 ratio, indicating that the classical RAS is overactivated, while the protective or non-classical branch of the RAS is suppressed. Seven RA patients demonstrated changes in CIMT, while eight patients developed arterial atherosclerotic plaques. These findings suggest that the classical RAS activation may contribute to the development of atherosclerosis (Braz et al., 2021).

The pathogenesis of diseases such as atherosclerosis is intricately linked to the interplay of Ang II and AT1R. When Ang II binds to AT1R, it triggers the activation of the JNK and ERK signaling pathways, leading to upregulated expression of early growth response factor 1 (Egr-1), which results in vasoconstriction, inflammation, oxidative stress, and increased expression of ICAM-1 and vascular cell adhesion molecule-1 (VCAM-1). These molecules facilitate the adhesion of monocytes to ECs, further driving the disease process. An *in vitro* study indicates that agomelatine can suppress the upregulation of AT1R expression in HUVECs and the human monocytic leukemia cell line THP-1 cells, and reduce the adhesion of monocytes to ECs, a process involved in the early stages of atherosclerosis. This suggests that agomelatine may alleviate Ang II-induced endothelial inflammation and monocyte adhesion by inhibiting AT1R activation and subsequent Egr-1 signaling, potentially exerting an anti-atherosclerotic effect (Hong et al., 2021). The renin-angiotensin-aldosterone system (RAAS) is vital for blood pressure regulation. In a population-based study, analysis of plasma samples reveals that in pathological states, increased membrane-bound angiotensin-converting enzyme 2 (mbACE2) helps convert Ang II to Ang 1-7, attempting to counteract the detrimental impacts of overactive RAAS. Overactivation of the RAAS results in increased expression of TNFα-converting enzyme (TACE), leading to more mbACE2 being cleaved into soluble ACE2 (sACE2). However, the elevation of sACE2 often reflects cardiac injury and disease state. Thus, elevated plasma sACE2 levels and the relative deficiency of mbACE2 may exacerbate the development of atherosclerosis and other coronary artery diseases (Hussain et al., 2021). Ang III, a metabolite of Ang II generated by aminopeptidase A (APA), serves as a significant endogenous AT2R agonist, influencing the kidneys and coronary arteries. Stimulating AT2R elicits a series of beneficial outcomes, including diuresis, vasodilation, and anti-inflammatory actions, which counteract the effects mediated by AT1R. In a study, the plasma concentrations of Angiotensin III and Angiotensin-converting enzyme are assessed among 44 healthy individuals and 84 patients with coronary atherosclerosis (CAS). The findings indicate that individuals exhibit notably reduced circulating angiotensin III levels, while APA levels are slightly decreased. These CAS patients are further categorized into two subgroups according to the degree of atherosclerosis. Compared to controls, the high-score group has lower levels of APA and Ang III, with a significant decrease in APA. These results imply that Ang III may offer a safeguarding effect against CAS (Yao et al., 2021). In summary, the RAS plays a crucial role in atherosclerosis.

**FIGURE 2:**
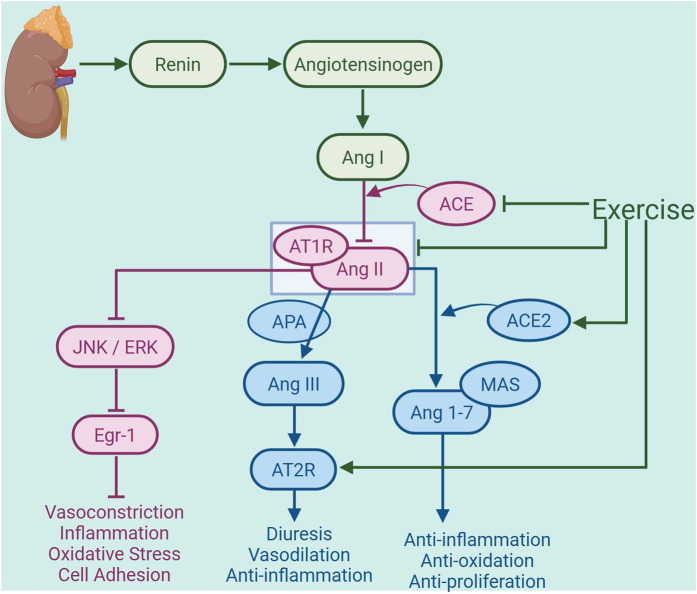
Exercise-regulated renin-angiotensin system (RAS) in amelioration of atherosclerosis. Under conditions of atherosclerosis, renin initiates the hydrolysis of angiotensinogen (ATG) to generate angiotensin I (Ang I), which is subsequently converted to angiotensin II (Ang II) by angiotensin-converting enzyme (ACE). This represents the activation of the classical arm of the renal RAS. Exercise inhibits the production of ACE and the binding of Ang II to the receptor AT1R, thereby reducing the activation of the JNK/ERK signaling pathway and suppressing early growth response factor 1 (Egr-1) expression, and consequently mitigating vas-oconstriction, inflammation, oxidative stress, and cell adhesion. Additionally, exercise can enhance the expression of AT2R in the heart. AT2R binds to Ang III, which is generated from Ang II by the action of aminopeptidase A (APA), thereby exerting diuretic, vasodilatory, and anti-inflammatory effects. Exercise also promotes the production of ACE2, which facilitates the conversion of Ang II into angiotensin-(1-7) [Ang 1-7], activating the non-classical pathway of the RAS. Ang 1-7 is a vasodilator peptide that exerts anti-inflammatory, antioxidative, and antiproliferative actions by binding to the receptor Mas, thereby alleviating the development of atherosclerosis. The graph was created with biorender.com (agreement number: HW28KV9DFS).

Exercise can alleviate atherosclerosis by activating the protective axis of the RAS. Medium-intensity treadmill exercise training leads to an increase of the activity RAS in visceral adipose tissue (VAT) of obese mice. After the exercise program, there is a marked reduction in Ang II and AT1R, as well as an elevation in Ang 1-7 levels, as demonstrated by the results. Additionally, the ratio of Ang II/Ang 1–7 decreases post-exercise training, which inhibits the classical branch of RAS (Alexandre-Santos et al., 2022). In a separate investigation, male Wistar rats were fed either a regular control diet (CON) or a high-fat regimen (HF) over 32 weeks to create an obesity model. the HF group exhibited reduced plasma ACE2 activity and a higher plasma ACE/ACE2 ratio compared to the CON group. Additionally, cardiac ACE activity and cardiac AT1R expression are elevated in the HF group. However, aerobic exercise at different intensities effectively reverses these outcomes. This suggests that exercise has the potential to mitigate RAS activation (Alexandre-Santos et al., 2020). Furthermore, recent studies indicate that resistance training can greatly lower Ang I and Ang II levels, fostering a transition of the renin-angiotensin system from the ACE/Ang II pathway to the ACE2/Ang 1-7 pathway in diabetic rats, as well as alleviate inflammation and enhance kidney function in diabetic mice. This suggests that exercise can suppress the classical arm of RAS and activate its protective branch (de Alcantara Santos et al., 2021). 10 weeks of treadmill endurance training elevate AT2R expression in the hearts of elderly rats, exerting cardioprotective effects (Raji-Amirhasani et al., 2018). This result suggests that exercise may activate the Ang III/AT2R axis, which remains to be further investigated. Consistent with the findings from animal studies, evidence in humans has demonstrated that exercise can reduce the risk of atherosclerosis by decreasing the expression of Ang II. A random effects meta-analysis finds that exercise training in human significantly reduced plasma Ang II concentration, which helps to reduce oxidative stress and inflammatory responses, thereby improving endothelial function to mitigate the risk of atherosclerosis (Baffour-Awuah et al., 2023). Consequently, exercise exerts an inhibitory effect on the classical arm of the RAS while simultaneously activating its non-classical arm, which may contribute to the mitigation of atherosclerosis (Figure 2).

However, several limitations should be considered when interpreting these findings. First, although both human and animal studies suggest a regulatory role of exercise on the RAS, the translational applicability of animal models to human pathophysiology remains uncertain. Additionally, most current evidence focuses on systemic RAS components, while the role of tissue-specific RAS, particularly in vascular beds, remains underexplored. Future studies should investigate the temporal and intensity-dependent effects of exercise on RAS signaling in different vascular contexts, and whether pharmacological modulation of RAS in combination with exercise confers additive benefits.

### 2.4 Effect of metabolites produced by exercise on alleviating atherosclerosis

Exercise induces profound metabolic adaptations that contribute to cardiovascular health, partly through the production and regulation of specific metabolites. These exercise-generated metabolites, including alpha-ketoglutarate (α-KG), lactate, and Lac-Phe, have emerged as important mediators in alleviating atherosclerosis. By modulating oxidative stress, inflammation, immune responses, and vascular function, these metabolites link exercise to improved vascular homeostasis and reduced atherogenesis. This section highlights recent advances elucidating how exercise-induced metabolites influence the progression of atherosclerosis.

#### 2.4.1 α-KG

**FIGURE 3:**
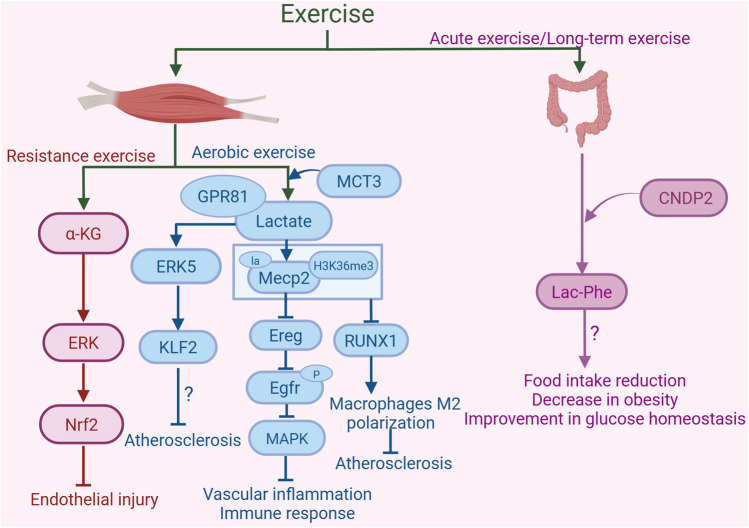
Exercise-induced metabolites in the protection against atherosclerosis. Resistance exercise enhances the synthesis and release of α-ketoglutarate (α-KG) in mouse muscle. α-KG activates nuclear factor erythroid 2-related factor 2 (Nrf2) by activating the ERK signaling pathway, inhibiting oxidative stress and mitochondrial dysfunction, thereby providing protective effects against endothelial damage. Aerobic exercise increases the production of lactate in skeletal muscle. Monocarboxylate transporter 3 (MCT3) is involved in the transport of lactate out of skeletal muscle. Lactate, by binding to the receptor GPR81, activates the ERK5 pathway to increase the expression of the arterial anti-atherosclerotic protective transcription factor Kruppel-like factor 2 (KLF2), thus playing a role in resisting atherosclerosis. Furthermore, lactate can activate lactylation of Mecp2 on lysine 271 in aortic ECs, subsequently inhibiting the expression of epidermal growth factor receptor (Egfr) ligand epiregulin (Ereg). This inhibition reduces the phosphorylation of Egfr, thereby blocking its downstream MAPK signaling pathway. These changes collectively alleviate vascular inflammation and immune responses. Additionally, exercise promotes the lactylation modification of Mecp2 at lysine 271, which facilitates the interaction of histone H3 trimethylation at Lys36 (H3K36me3) in macrophages of the aortic root plaque. This interaction leads to the demethylation of H3K36me3, suppression of the expression of RUNX family transcription factor 1 (RUNX1), and promotion of M2 macrophage polarization, thereby alleviating atherosclerosis. Acute exercise and long-term exercise training promote the production of Lac-Phe in macrophages and intestinal epithelial cells, with CNDP2 being a major Lac-Phe biosynthetic enzyme. The increase in Lac-Phe is associated with reduced food intake, decreased obesity, and improved glucose homeostasis, which may contribute to the alleviation of atherosclerosis. The graph was created with biorender.com (agreement number: KL28KV9IFU).

The substantial accumulation of the intermediate metabolite α-KG in the tricarboxylic acid cycle (TCA) is a metabolic signature of resistance training (Peng et al., 2021). Endurance exercise can induce a rise in serum α-KG levels in both normally fed and HFD-fed mice. In contrast to endurance exercise, resistance exercise markedly elevates serum α-KG concentrations, suggesting that the extent of exercise-induced increases in serum α-KG is dependent on the type of exercise. Moreover, this study indicates the type of the exercise, rather than its intensity, plays a primary role in increasing serum α-KG levels (Yuan et al., 2020). Additionally, studies in mice have demonstrated that α-KG activates nuclear factor erythroid 2-related factor 2 (Nrf2) by activating the ERK signaling pathway in the endothelium of the thoracic aorta to counteract oxidative stress and mitochondrial impairment, thereby providing protective effects against endothelial damage induced by hyperlipidemia. Furthermore, α-KG can increase NO levels and the expression of eNOS in ECs treated with palmitic acid, which facilitates vasodilation. α-KG also suppresses the secretion of endothelin-1 (ET-1) in PA-induced ECs to attenuate vasoconstriction. This suggests that an increase in α-KG may inhibit endothelial injury in atherosclerosis (Cheng D. et al., 2023). In conclusion, exercise may stimulate the production of α-KG, which has the potential to mitigate atherosclerosis (Figure 3).

#### 2.4.2 Lactate

Lactate, primarily produced through glycolysis, is crucial for maintaining cardiovascular system homeostasis (Lactate helps in tissue repair, 2023). Under physiological conditions, lactate contributes to maintaining cardiovascular homeostasis by providing energy and regulating signals. Lactate may reduce atherosclerosis risk via multiple mechanisms (Wu et al., 2023). A study utilizes HUVECs to simulate the inflammatory response of ECs under oscillatory shear stress (OSS), constructing an early atherosclerosis model. The research reveals that lactate, the endogenous ligand of G protein-coupled receptor 81 (GPR81), promotes the production of the atheroprotective transcription factor kruppel-like factor 2 (KLF2) via the extracellular signal-regulated kinase 5 (ERK5) signaling pathway, which is crucial for preventing atherosclerosis. KLF2 plays a key role by inhibiting the expression of adhesion molecules like VCAM-1 and E-selectin, thereby reducing monocyte adhesion to ECs and protecting the vascular endothelium. However, ox-LDL exposure significantly downregulates KLF2 expression, counteracting this protective effect and promoting atherosclerosis. This shows that lactate, by activating GPR81 and its downstream pathways, exerts anti-atherosclerotic effects, but this process is impeded by ox-LDL-induced KLF2 downregulation (Sun et al., 2019). Another study indicates that inhibition of MCT3 impairs the transport of lactate out of skeletal muscle, potentially promoting SMCs proliferation and facilitating the development of atherosclerosis. This suggests a role for MCT3 in the process of lactate-mediated alleviation of atherosclerosis (Wu et al., 2023).

Exercise can alleviate atherosclerosis by increasing lactate production. A study discovers that 14 days of treadmill regimen increases lactate concentrations in the serum, liver, and muscles of mice (Yan et al., 2024). An 8-week regimen of treadmill exercise leads to elevated serum lactate concentrations in ApoE^−/−^ mice fed with HFD, activated the lactylation modification of Mecp2 at lysine 271 in aortic ECs, and subsequently suppressed the expression of the epidermal growth factor receptor (Egfr) ligand epiregulin (Ereg). This suppression reduces the phosphorylation of Egfr, thereby blocking its downstream MAPK signaling pathway, leading to decreased expression of VCAM-1, ICAM-1, MCP-1, IL-1β, IL-6, and an increased level of eNOS. These changes collectively alleviate vascular inflammation and immune responses, thus inhibiting the progression of atherosclerosis (Wang et al., 2023b). Interestingly, exercise (moderate aerobic treadmill training) promotes the lactylation modification of Mecp2 at lysine 271 in macrophages of the aortic root plaque in ApoE^−/−^ mice by increasing blood lactate concentration. This modification facilitates the interaction between Mecp2 and the histone modification histone H3 trimethylation at Lys36 (H3K36me3). As a result, it leads to the demethylation of H3K36me3, suppressing the expression of RUNX family transcription factor 1 (RUNX1) and promoting the polarization of pro-resolving M2 macrophages. Consequently, this reduces the atherosclerotic lesion area, shrinks the necrotic core, and increases collagen content, thereby enhancing plaque stability (Chen L. et al., 2024). Therefore, exercise may lead to upregulation of lactate levels and lactylation modification, thereby alleviating the progression of atherosclerosis (Figure 3).

Adipose inflammation and immune responses may contribute to the atherosclerotic process (Mazitova et al., 2023). It is shown that lactate signaling via GPR81 in monocytes/macrophages suppresses pro-inflammatory responses in adipose tissue (Hoque et al., 2014; Certo et al., 2022). Interestingly, recent evidence has unveiled a paradoxical role of lactate in the regulation of adipose immune responses during obesity. While lactate is often considered an immunosuppressive metabolite, it has been shown that monocarboxylate transporter 1 (MCT1), a key facilitator of lactate transport across the plasma membrane, plays a crucial role in promoting CD8^+^ T cell proliferation and infiltration into adipose tissue under obesogenic conditions. Specifically, MCT1 expression is markedly upregulated upon CD8^+^ T cell activation, supporting a metabolic switch toward aerobic glycolysis. Genetic ablation of MCT1 in T cells leads to impaired CD8^+^ T cell proliferation, a shift in energy metabolism toward oxidative phosphorylation, and a notable reduction in effector T cell accumulation within epididymal white adipose tissue (eWAT). This alteration is accompanied by reduced expression of pro-inflammatory cytokines and adipogenic genes, smaller adipocytes, and increased markers of adipose tissue browning, ultimately attenuating obesity-associated inflammation (Macchi et al., 2022a). Therefore, lactate plays complicated roles in regulating adipose inflammation and immune responses, thereby affecting the progression of atherosclerosis.

#### 2.4.3 Lac-Phe

Beyond the aforementioned exercise metabolites, exercise enhances Lac-Phe synthesis, a circulating signaling metabolite that curbs appetite and reduces obesity risk. The production of Lac-Phe autonomously increases in macrophages and intestinal epithelial cells in a cytosolic non-specific dipeptidase 2 (CNDP2)-dependent manner, where CNDP2 is a major Lac-Phe biosynthetic enzyme (Xiao et al., 2023). Findings suggest that both acute exercise and long-term exercise training in mice result in a notable elevation of plasma Lac-Phe concentrations. In mice, this surge in Lac-Phe is linked to a lower food consumption, a decline in obesity rates, and enhanced maintenance of glucose balance. Furthermore, global CNDP2 gene knockout mice exhibit a deficiency in Lac-Phe, which results in heightened food consumption and subsequent weight gain. In humans, post-exercise plasma levels of Lac-Phe have been observed to rise markedly and remain elevated, suggesting that Lac-Phe is crucial for regulating metabolic regulation of energy balance, particularly in reaction to exercise (Li VL. et al., 2022). Studies have shown that metformin promotes the biosynthesis of Lac-Phe. Elevated Lac-Phe levels decrease food consumption and body mass (Xiao et al., 2023). Notably, participants treated with metformin in the Multi-Ethnic Study of Atherosclerosis (MESA) study exhibit significantly elevated Lac-Phe levels compared to non-metformin users (Li et al., 2024). Additionally, a significant stepwise rise in Lac-Phe levels is detected as plasma metformin concentrations increased across quartiles. This points to a positive, dose-dependent relationship between Lac-Phe levels in the plasma and the levels of metformin. Such findings imply that elevated Lac-Phe levels could play a role in mitigating atherosclerosis (Xiao et al., 2024). In general, exercise may increase the level of Lac-Phe to alleviate atherosclerosis (Figure 3).

### 2.5 Effect of exercise-regulated gut microbiota in alleviating atherosclerosis

Gut microbiota dysbiosis promotes the progression of atherosclerosis by generating pro-inflammatory metabolites that trigger vascular inflammation and plaque instability. Exercise has been shown to modulate gut microbiota composition, reduce inflammatory mediators, and increase beneficial bacterial populations, thereby protecting vascular health and attenuating the progression of atherosclerosis. This section reviews recent advances in understanding how exercise-induced regulation of gut microbiota contributes to the alleviation of atherosclerosis.

Studies had shown that a HFD can disrupt gut microbial balance, linked to the development of atherosclerosis (Xu et al., 2022). A recent study utilized 16S rRNA sequencing to examine the gut microbiota composition in HFD-fed ApoE^−/−^ mice. The findings reveal that these mice exhibited diminished microbial diversity when compared to their healthy counterparts, with a notable rise in the proportion of Firmicutes and a corresponding decline in *Bacteroidetes* (Zhang Y. et al., 2021). Under the pathological conditions of atherosclerosis, increased production of the metabolic product lipopolysaccharide (LPS) by the gut microbiota can increase intestinal permeability, allowing bacteria and LPS to enter the bloodstream. Upon binding to TLR4, LPS activates the downstream signaling pathway that is myeloid differentiation primary response 88 (MyD88)-dependent, resulting in increased production of inflammatory mediators that promote inflammatory responses, thereby facilitating the formation and development of atherosclerotic plaques (Yang S. et al., 2021). In addition to LPS, under the pathological conditions of atherosclerosis, increased production of the metabolic product trimethylamine N-oxide (TMAO) by the gut microbiota can promote fosters leukocyte adherence and movement toward the vascular wall by upregulating the expression of cell adhesion molecules in ECs, as well as activate the NLR family pyrin domain containing 3 (NLRP3) inflammasome to enhance inflammatory responses, thereby destabilizing atherosclerotic plaques (Wang C. et al., 2024). Moreover, other metabolites produced by the gut microbiota also undergo changes under atherosclerotic conditions, such as decreased levels of SCFAs and increased levels of branched-chain amino acids (BCAAs) and aromatic amino acids (AAAs). These findings indicate that changes in the levels of metabolites produced by the gut microbiota under atherosclerotic conditions may weaken the stability of atherosclerotic plaques and worsen the advancement of atherosclerosis (Shen et al., 2021). These results indicate that addressing gut microbiota dysbiosis may offer a promising strategy for preventing atherosclerosis.

**FIGURE 4:**
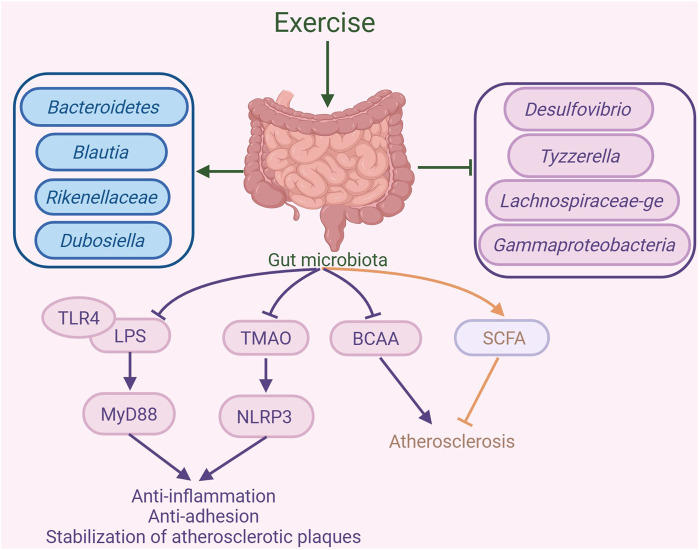
Exercise-mediated gut microbiota changes in the amelioration of atherosclerosis. Exercise can alter the composition of the gut microbiota, increasing the abundance of *Bacteroidetes, Blautia, Rikenellaceae*, and *Dubosiella*, while decreasing the abundance of *Desulfovibrio, Tyzzerella, Lachnospiraceae-ge*, and *Gammaproteobacteria*. By regulating the gut microbiota, exercise inhibits the binding of lipopolysaccharide (LPS) and Toll-like receptor 4 (TLR4), suppressing the MyD88 signaling pathway, which in turn suppresses inflammatory responses and the formation of atherosclerotic plaques. Additionally, exercise inhibits trimethylamine N-oxide (TMAO) expression and the NLR family pyrin domain-containing 3 (NLRP3) inflammasome to suppress inflammatory responses, thereby promoting the stability of atherosclerotic plaques. Furthermore, exercise can inhibit branched-chain amino acids (BCAAs) production and promote short-chain fatty acid (SCFA) to exert anti-atherosclerotic effects. The graph was created with biorender.com (agreement number: UF28KV9P6Z).

Exercise can change the makeup and function of gut microbiota, resulting in a series of positive outcomes. In a study, diabetic patients engaged in either short-term high-intensity interval training or moderate-intensity continuous training (MICT) thrice weekly for 2 weeks. The results show that both training modalities lead to a decrease in inflammatory biomarkers, including TNFα and endotoxin-binding protein, and triggered changes in the gut flora composition. Specifically, there is a notable increase in the proportion of *Bacteroidetes*, a corresponding decrease in the *Firmicutes*-to-*Bacteroidetes* ratio, and a reduction in the presence of *Clostridium* and *Blautia* genera. Exercise training may help lower the risk of by modifying gut microbiota composition and decreasing inflammatory markers (Motiani et al., 2020). A 12-week regimen of concurrent strength and endurance training is found to reduce the abundance of obesity-associated gut microbiota. Notably, it leads to a substantial reduction in the prevalence of the *Proteobacteria* phylum and the *Gammaproteobacteria* class, in turn diminishing gut inflammation. Concurrently, the exercise increases the prevalence of advantageous bacterial genera like *Blautia*, *Dialister*, and *Roseburia*, which aids in maintaining gut health and reducing inflammation. Additionally, in the atherosclerotic mouse model induced by a WD, dysbiosis leads to a significant increase in inflammation-associated microbiota, such as *Desulfovibrio*, *Tyzzerella*, and *Lachnospiraceae-ge*. Following endurance exercise intervention, the abundance of these inflammation-related microbiota is significantly reduced, while that of beneficial microbiota, such as Rikenellaceae and *Dubosiella*. These microbiotas are capable of producing SCFAs (particularly propionate and butyrate) in the gut, which mitigate the inflammatory response in atherosclerosis by enhancing gut barrier function and inhibiting inflammatory signaling pathways (Huang et al., 2022). In summary, these results indicate that exercise can help adjust gut microbiota imbalances, potentially alleviating atherosclerosis (Figure 4).

### 2.6 Effect of exercise-modulated cell death on ameliorating atherosclerosis

**FIGURE 5:**
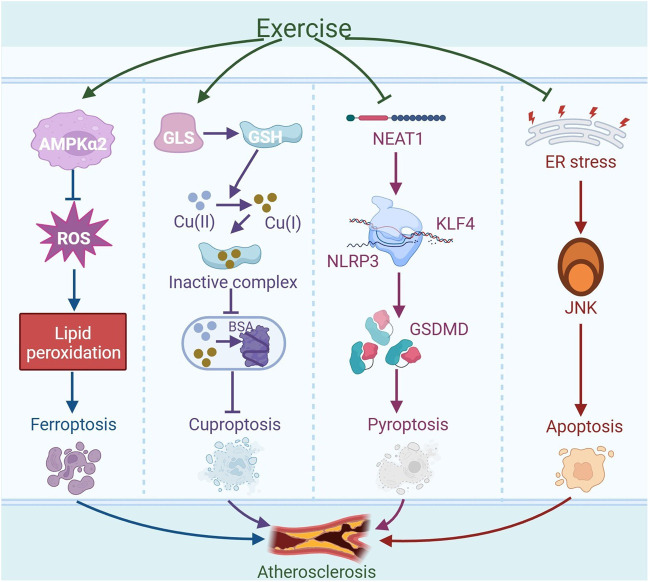
Exercise-regulated cell death in amelioration of atherosclerosis. Exercise may mitigate ferroptosis by activating AMPKα2, reducing oxidative stress, and inhibiting lipid peroxidation, ultimately alleviating atherosclerosis. Exercise can enhance the expression of glutaminase (GLS) to produce antioxidant glutathione (GSH), which can reduce Cu (II) to Cu (I). Subsequently, it forms inactive complexes by binding with Cu (I), thereby preventing copper ions from disrupting the normal structure and function of bovine serum albumin (BSA) within cells, thus inhibiting cuproptosis and potentially mitigating atherosclerosis. Moreover, exercise can reduce the expression of nuclear paraspeckle assembly transcript 1 (NEAT1) in the thoracic aorta of atherosclerotic mice. The downregulation of NEAT1 disrupts the transcriptional activation of the downstream gene NLRP3 by kruppel-like factor 4 (KLF4), leading to decreased expression of NLRP3. This reduction in NLRP3 expression results in a decrease in the expression of the NLRP3-mediated pyroptosis-associated gene GSDMD, which contributes to the inhibition of EC pyroptosis and thus alleviates atherosclerosis. In addition, exercise may reduce the activation of the JNK signaling pathway induced by endoplasmic reticulum (ER) stress, thereby inhibiting apoptosis and alleviating atherosclerosis. The graph was created with biorender.com (agreement number: KB28KV9VWL).

Exercise has the ability to modulate various forms of cell death, including ferroptosis, cuproptosis, pyroptosis, and apoptosis. The alterations in these cell death processes induced by exercise may alleviate atherosclerosis, as illustrated in Figure 5.

#### 2.6.1 Ferroptosis

Ferroptosis is a mechanism of cell death that is non-apoptotic and marked by iron-dependent peroxidation of membrane lipids (Dixon and Olzmann, 2024). Iron overload accelerates the development of atherosclerosis by enhancing oxidative and inflammatory responses (Lin et al., 2022). Ox-LDL can trigger ferroptosis in macrophages, ECs and VSMCs. Glutathione peroxidase 4 (GPX4) is an important antioxidant enzyme, when its activity diminishes, results in lipid peroxide accumulation and initiates ferroptosis (You Z. et al., 2023). Cytokines like IL-1β and IL-18, secreted in ferroptosis, can intensify the inflammatory reaction and contribute to the advancement of atherosclerosis (Lu et al., 2024). Additionally, activation of the Nrf2 signaling cascade in ApoE^−/−^ mice inhibits ferroptosis in macrophages, thereby reducing oxidative stress and inflammatory responses and alleviating atherosclerosis (Tao et al., 2024). Aside from macrophage ferroptosis, ferroptosis also occurs in ECs. Oxidized phosphatidylcholine (PGPC), a component of atherosclerotic plaques, can induce ferroptosis in ECs by binding to its receptor cluster of differentiation 36 (CD36) and increasing the levels of fatty acid-binding protein 3 (FABP3). This ferroptosis induces endothelial dysfunction and accelerates atherosclerosis development. Moreover, interventions using the ferroptosis inhibitor ferrostatin-1 (Fer-1) or targeting FABP3 can alleviate PGPC-induced ferroptosis in ECs, improve endothelial function and mitigate atherosclerosis in ApoE^−/−^ mice (Chen S. et al., 2024). In addition, Fer-1 can reduce iron accumulation in VSMCs induced by ox-LDL, and activate the Nrf2/ferroptosis suppressor protein 1 (FSP1) pathway to enhance intrinsic defenses against lipid peroxidation in the cells, thereby inhibiting ferroptosis in VSMCs and reducing atherosclerotic lesions in ApoE^−/−^ mice (You J. et al., 2023).

Exercise programs might be crucial in the mitigation and management of conditions associated with ferroptosis. Endurance exercise can reduce ROS production and inhibit lipid peroxidation by activating AMPKα2 in mouse cardiomyocytes, thereby suppressing ferroptosis in cardiomyocytes induced by doxorubicin (Wang L. et al., 2024). In aged rats, long-term exercise activates the Nrf2 signaling pathway, which boosts the production of antioxidant proteins associated with ferroptosis in skeletal muscle, such as GPX4 and solute carrier family seven member 11 (SLC7A11), thereby augmenting the antioxidant capacity of skeletal muscle and reducing ferroptosis (Wang ZZ. et al., 2023). Moreover, moderate-intensity treadmill exercise has been shown to significantly reduce serum levels of ferrous iron (Fe^2+^), while concurrently increasing the GSH/GSSG ratio in traumatic brain injury (TBI) models. These changes reflect an amelioration of iron overload and a restoration of redox homeostasis, collectively demonstrating the potent antioxidant and anti-ferroptotic effects of physical exercise during the chronic phase of TBI recovery (Chen J. et al., 2023). In summary, exercise may alleviate atherosclerosis by inhibiting ferroptosis.

#### 2.6.2 Cuproptosis

Cuproptosis represents a novel form of cell death that does not follow the traditional apoptotic pathway, distinguished by its dependence on copper ions and the modulation of mitochondrial respiration. Studies in the realm of ASCVD have indicated a direct link between heightened serum copper concentrations and the onset and worsening of atherosclerosis (Rose and Rusu, 2024). Copper overload exhibits toxic effects, resulting in cellular harm and potential cell death. An abundance of copper triggers a reduction process and fosters cell death by encouraging the abnormal oligomerization of copper-dependent, lipoylated proteins in the tricarboxylic acid (TCA) cycle, while simultaneously diminishing the concentration of iron-sulfur cluster proteins. Increased copper ion levels are found in the serum and lesions of people with atherosclerosis, and excessive deposition of copper ions in lysosomes can lead to the death of HUVECs. Furthermore, cuproptosis-related genes ferredoxin 1 (FDX1), solute carrier family 31 member 1 (SLC31A1), and glutaminase (GLS) are crucial in the onset and advancement of atherosclerosis. Specifically, FDX1 and SLC31A1 show increased expression in atherosclerotic plaques, while GLS exhibits decreased levels. FDX1 has the ability to directly interact with copper ions present in plaques, thereby triggering protein-induced toxic stress. SLC31A1, a copper transporter protein, is responsible for importing copper from the circulation system into the plaques. The upregulation of SLC31A1 in macrophages present in atherosclerotic plaques implies that it could contribute to the accumulation of copper within these lesions, leading to increased production of ROS, thereby reducing plaque stability. GLS, by converting glutamine into glutamate, boosts intracellular GSH synthesis to counteract ROS-induced harm and shield cells from copper-induced toxicity (Cui et al., 2023). Studies have demonstrated that GSH can inhibit copper-induced aggregation of bovine serum albumin (BSA) across various cell lines, including BEAS-2B, and Hepa1-6. Since Cu (II) and GSH cannot coexist, GSH initially converts Cu (II) into Cu (I), and subsequently binds the reduced form of the metal to form an inactive complex. This process prevents copper ions from disrupting the normal structure and function of proteins, thereby maintaining protein homeostasis within the cell. These findings indicate that, in addition to its established antioxidant functions, GSH is also capable of preventing copper-induced protein aggregation, protecting cells from cuproptosis (Saporito-Magriñá et al., 2018). An 8-week treadmill workout regimen was shown to enhance GLS activity within T lymphocytes in rats. Additionally, both high-intensity interval training (HIIT) and MICT for 6 weeks enhance the generation of GSH in the skeletal muscle of ApoE^−/−^ mice, which increases GSH levels and helps to alleviate oxidative damage (Wang et al., 2021). Another study finds that when human coronary artery endothelial cells (HCAECs) are stimulated with high concentrations of copper (50 μM CuCl2), the process of cuproptosis is activated, leading to the death or dysfunction of HCAECs. Moreover, NLRP3 activation is linked to increased copper concentrations, suggesting that cuproptosis may trigger NLRP3 to advance atherosclerosis progression (Wang M. et al., 2023). Overall, cuproptosis promotes the progression of atherosclerosis, and exercise may alleviate atherosclerosis by inhibiting cuproptosis.

Critically, while current studies have begun to elucidate the potential role of cuproptosis in atherosclerosis and its modulation by exercise, several gaps remain. Many findings are based on *in vitro* models or animal studies, with limited validation in human subjects, which restricts their translational relevance. Moreover, the mechanisms by which exercise alters copper homeostasis or cuproptosis-related gene expression in vascular cells remain incompletely defined. For instance, although enhanced GLS activity and GSH production are observed after exercise, it is unclear whether these changes are sufficient to prevent cuproptosis *in vivo* or merely reflect a general antioxidant adaptation. In addition, most studies do not differentiate between exercise modalities, durations, or intensities when evaluating their effects on cuproptosis-related pathways. Therefore, while the concept of exercise-mediated inhibition of cuproptosis is compelling, further mechanistic and longitudinal studies are needed to clarify causality and optimize exercise prescriptions for atherosclerosis prevention through this pathway.

#### 2.6.3 Pyroptosis

Pyroptosis represents a distinct variety of inflammatory programmed cell death, marked by the activation of caspase-1 in a process reliant on the NLRP3 inflammasome. This activation is essential for the maturation of the Gasdermin D precursor (GSDMD), as well as IL-1β and IL-18. In a study, GSDMD was found to be activated within atherosclerotic plaques of humans and mice. Moreover, the pyroptosis instigated by macrophage-derived GSDMD is pivotal in the development of these plaques. Genetic deletion of GSDMD (GSDMD^−/−^) mitigates the size of atherosclerotic lesions in HFD-fed ApoE^−/−^ mice. GSDMD not only drives mitochondrial perforation but also triggers the release of mitochondrial DNA (mtDNA) (Zhu et al., 2022). This release subsequently triggers the STING-IRF3 (interferon regulatory factor 3)/NF-κB axis, and simultaneously promotes the activation of cGAS-STING-TBK1(TANK-binding kinase 1)-IRF3 and macrophage migration, which promotes inflammation and macrophage apoptosis (Fan et al., 2024). In summary, multiple pathways promote pyroptosis in macrophages and ECs during atherosclerosis.

Studies find that exercise can alleviate cellular pyroptosis. 12 weeks of treadmill training has been shown to lower the N6-methyladenosine (m6A) methylation status of nuclear paraspeckle assembly transcript 1 (NEAT1) in the thoracic aorta of atherosclerotic mice, leading to decreased expression of NEAT1. The downregulation of NEAT1 disrupts the transcriptional activation of NLRP3 by Kruppel-like factor 4 (KLF4), resulting in reduced expression of NLRP3. NLRP3 is a key pyroptotic protein, and the decrease in its expression contributes to the inhibition of EC pyroptosis, thereby alleviating atherosclerosis (Yang et al., 2023). Importantly, aerobic exercise notably diminished the levels of pyroptosis-associated markers, such as NLRP3, caspase-1, GSDMD, IL-1β, and IL-18, in the aortas of atherosclerotic mice by modulating the NLRP3 inflammasome (Li XH. et al., 2022). In addition, exercise training inhibited inflammasome activation and pyroptosis by shifting microglial polarization from the pro-inflammatory M1 to the anti-inflammatory M2 type (Liu et al., 2022). Therefore, exercise may alleviate atherosclerosis by inhibiting the expression of pyroptosis-related markers.

#### 2.6.4 Apoptosis

Apoptosis is a controlled process of cell death executed by specific enzymes such as caspase-3 and caspase-7. Once activated within the cell, these enzymes proceed to cleave a range of intracellular targets, resulting in the orderly disassembly of cellular structures and the formation of apoptotic bodies. These structures are eventually engulfed and removed by phagocytic cells (Ai et al., 2024). In atherosclerosis, apoptosis leads to the death of macrophages. As the lesion progresses, apoptotic macrophages that are not promptly cleared may undergo secondary necrosis, increasing inflammatory responses and expanding the necrotic core, potentially causing plaque instability and rupture, thus progressing the condition (De Meyer et al., 2024). It is found that systemic deletion of the neuropeptide receptor C (NPRC) in atherosclerotic mice indirectly inhibits NF-κB activity by triggering the PKA signaling cascade. This mechanism leads to a decrease in the expression of apoptosis-associated genes, including caspase-3 and caspase-7, thereby inhibiting the apoptosis of ECs and attenuating the severity of atherosclerosis (Cheng C. et al., 2023; Jiang et al., 2023). In addition, the oral pathogen *P. gingivalis* (*Porphyromonas gingivalis*) has the capacity to interact directly with toll-like receptor 2 (TLR2) on host cells. This interaction amplifies the NF-κB signaling pathway, thereby stimulating the production of apoptosis-related proteins such as caspase-3 and poly (ADP-ribose) polymerase (PARP). Consequently, this results in an elevated rate of apoptosis in SMCs, thereby intensifying the progression of atherosclerosis (Xie et al., 2023; Liang et al., 2024). These results suggest that cell apoptosis mediated through various pathways may contribute to the progression of atherosclerosis.

Exercise training can reduce age-related apoptosis of cardiac cells by lowering the proportion of pro-apoptotic protein Bax in relation to anti-apoptotic protein Bcl-2 in cardiac cells, thereby slowing down cardiac remodeling and functional decline. In a mouse study, aerobic exercise inhibits cardiomyocyte apoptosis by inhibiting ER stress-induced apoptotic signaling pathways, such as including the downregulation of critical proteins like CCAAT/enhancer-binding protein CHOP, JNK, and caspase-12, which may contribute to the alleviation of atherosclerosis (Cai et al., 2020). In summary, exercise can enhance the levels of anti-apoptotic factors while suppressing the expression of pro-apoptotic molecules, which ultimately help to dampen the advancement of atherosclerosis.

### 2.7 Effect of exercise‐regulated microRNAs on alleviating atherosclerosis

The microRNAs (miRNAs) play a crucial regulatory role in atherosclerosis. Exercise modulates miRNAs expression to affect inflammation, lipid metabolism, and cellular functions, thereby slowing the progression of atherosclerosis. This section briefly reviews recent advances on exercise-regulated miRNAs in alleviating atherosclerosis.

The miRNAs negatively play a crucial role in downregulating gene expression by attaching to the 3′-untranslated regions (3′UTR) of target mRNAs. This interaction leads to the breakdown of the mRNA and/or hampers the efficiency of translation of the gene products. A notable characteristic of miRNAs is their pleiotropic nature, meaning a single miRNA molecule can regulate multiple mRNAs across various biological pathways. And conversely, multiple miRNAs can target a single mRNA. As key regulators within the post-transcriptional control network, miRNAs significantly contribute to the pathogenesis of atherosclerosis (Raskurazhev et al., 2022). A randomized trial included 31 patients with CAD who have undergone percutaneous coronary intervention (PCI). Following PCI, plasma expression levels of miRNAs are assessed subsequent to either aerobic interval training or moderate continuous training. Following the exercise regimen, there is a noticeable reduction in the levels of miR-93-5p and miR-451a, coupled with a surge in miR-146a-5p levels, which corresponded with a decline in the coronary plaque load. These miRNAs could potentially serve as promising biomarkers for CAD and its responsiveness to exercise.

Exercise modulates the expression of miRNAs, which subsequently play roles in the progression of atherosclerosis via diverse molecular pathways (Table 1). Exercise intervention significantly reduced miR-23b and miR-151-5p expression, indicating their potential involvement in atherosclerosis pathogenesis. Conversely, the levels of miR-362-3p, miR-324-3p, miR-140-5p, miR-30e and miR-532-5p are found to be upregulated following exercise, suggesting that these particular miRNAs might be instrumental in regulating vascular health and could be pivotal in inhibiting the progression of atherosclerosis (Radom-Aizik et al., 2014). For example, in a cellular experiment, miR-23b has been shown to augment the inflammatory response triggered by ox-LDL in macrophages via the A20/NF-κB signaling axis, thereby facilitating the progression of atherosclerosis (He et al., 2020). In another cellular study, miR-362-3p is shown to directly bind to and negatively regulate the expression of a disintegrin and metalloproteinase with thrombospondin motifs 1 (ADAMTS1), which suppresses VSMCs proliferation and migration in atherosclerosis. This research shows that the miR-362-3p mimic elevates miR-362-3p levels in VSMCs, subsequently causing a marked decrease in ADAMTS1 mRNA and protein expression. Overexpression of ADAMTS1 through plasmid transfection notably mitigated the suppressive effects of miR-362-3p on VSMC proliferation, cell cycle progression, and migration. The research indicates that miR-362-3p is integral to the pathogenesis of atherosclerosis by regulating ADAMTS1 expression (Li et al., 2017). miR-324-3p targets genes associated with inflammation and apoptosis (such as NOS1, BCL2L11, p53), the synthesis of NO, and the release of mitochondrial cytochrome c, which collectively suppresses cellular apoptosis and inflammatory responses, and alleviating the progression of atherosclerosis (Bhansali et al., 2023). miR-140-5p inhibits TLR4 mRNA expression through 3′UTR binding, thereby reducing macrophage lipid accumulation, oxidative stress, and apoptosis associated with atherosclerosis (Liu et al., 2021). In ApoE^−/−^ mice, the use of miR-30e inhibitor elevates levels of insulin-like growth factor 2 (IGF2) in both the aorta and liver. Furthermore, it markedly bolstered the expression of the osteogenic marker OPN protein and promoted calcium deposition in the aortic valve. This indicates that miR-30e inhibits osteogenic program and vascular calcification by targeting IGF2, thereby alleviating the development of atherosclerosis (Ding et al., 2015). MiR-532-5p alleviates ox-LDL-induced EC injury and ameliorates atherosclerosis by suppressing the expression of Rho-associated protein kinase 2 (ROCK2) (Li et al., 2023). The target of miR-151-5p that may alleviate atherosclerosis is unclear and requires further study. After a 12-week of swimming regimen, notable increases in miR-492 and decreases in resistin levels are observed in the serum of ApoE^−/−^ mice. Meanwhile, upregulating expression of miR-492 and downregulating expression of resistin in HUVECs improve endothelial insulin resistance and delayed the progression of atherosclerosis by suppressing the phosphorylation of signal transducer and activator of transcription 3 (STAT3), the activity of suppressor of cytokine signaling (SOCS) and P-selectin (Cai et al., 2018; Ying et al., 2014). Aerobic exercise has the potential to upregulate the level of miR-146a in serum, which may mitigate vascular tumor necrosis factor receptor-associated factor (TRAF) and TLR4 pathways, thereby attenuating vascular inflammation and damage in atherosclerosis (Wu et al., 2014).

**TABLE 1:** Roles of microRNAs in the alleviation of atherosclerosis through exercise.

microRNAs	Changed by exercise	Target(s)	Role(s) in atherosclerosis
miR-93-5p	↓	^?^	Promotion of atherosclerosis^a^
miR-451a	↓	^?^	Promotion of atherosclerosis^a^
miR-146a-5p	↑	^?^	Inhibition of atherosclerosis^a^
miR-23b	↓	A20	Promotion of inflammation
miR-151-5p	↓	^?^	Promotion of atherosclerosis^a^
miR-362-3p	↑	ADAMTS1	Inhibition of VSMC proliferation and migration
miR-324-3p	↑	NOS1	Inhibition of macrophage apoptosis
		BCL2L11	Suppression of inflammatory response
		p53	Stabilization of atherosclerotic plaques
miR-140-5p	↑	TLR4	Inhibition of macrophage apoptosis
			Reduction of macrophage lipid accumulation and oxidative stress
miR-30e	↑	IGF2	Inhibition of osteogenic program and vascular calcification
miR-532-5p	↑	ROCK2	Mitigation of endothelial damage
miR-492	↑	Resistin	Improvement of endothelial insulin resistance
miR-146a	↑	TLR4	Mitigation of vascular inflammation and damage

^a^ The detailed mechanism is not known.

^?^ The target gene is not known.

**FIGURE 6:**
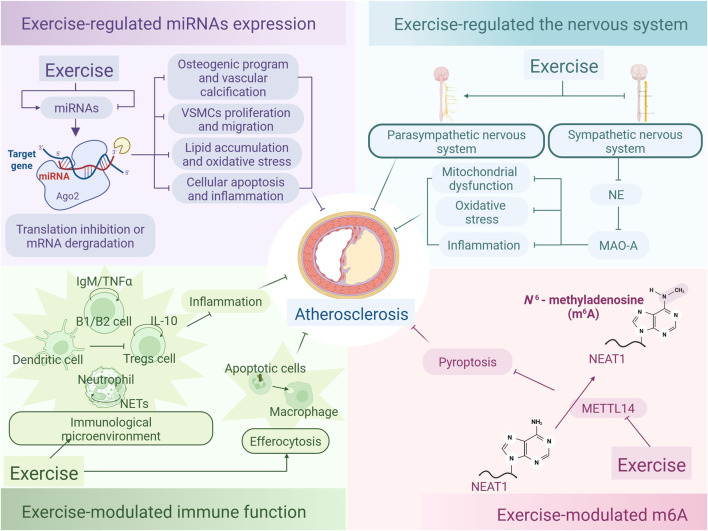
Exercise-regulated miRNAs, nervous system, immune cells, and m6A in the alleviation of atherosclerosis. Exercise alleviates atherosclerosis by modulating the expression of miRNAs, which target genes to promote translational repression or degradation, thereby inhibiting osteogenic programs and vascular calcification, VSMCs proliferation and migration, lipid accumulation and oxidative stress, as well as cell apoptosis and inflammation. Additionally, exercise mitigates atherosclerosis by promoting parasympathetic activity and inhibiting sympathetic activity, thereby suppressing the expression of norepinephrine (NE) and MAO-A, and reducing mitochondrial dysfunction, oxidative stress, and inflammation. Furthermore, exercise may maintain the homeostasis of the immune microenvironment by inhibiting the release of neutrophil extracellular traps (NETs) from neutrophils and the production of TNFα by B2 cells, promoting the generation of natural IgM antibodies from B1 cells and the secretion of IL-10 by Tregs, as well as suppressing the inhibitory effects of dendritic cells (DCs) on Tregs, thereby alleviating atherosclerosis. Exercise also diminishes METTL14 activity and expression in the thoracic aorta of atherosclerotic mice. This inhibition reduces METTL14-mediated m6A modification of NEAT1 mRNA, leading to lower NEAT1 expression, which ultimately suppresses endothelial cell pyroptosis and alleviates atherosclerosis. The graph was created with biorender.com (agreement number: QK28KVA0MB).

In summary, exercise has the potential to alleviate atherosclerosis through upregulating or downregulating the expression of different miRNAs (Figure 6).

### 2.8 Effect of exercise-modulated nervous system on ameliorating atherosclerosis

The nervous, immune, and cardiovascular systems are interconnected networks that respond to and interact with each other, jointly regulating cardiovascular function (Mohanta et al., 2023). Autonomic nervous system dysfunction, especially increased sympathetic activity, promotes inflammation, oxidative stress, and endothelial dysfunction, accelerating atherosclerosis. Exercise may improve vascular function and slow disease progression by enhancing parasympathetic activity and inhibiting sympathetic overactivation. This section reviews recent advances on exercise-mediated nervous system regulation in alleviating atherosclerosis.

Atherosclerosis is characterized by the development of immune cell clusters known as arterial tertiary lymphoid organs (ATLOs), which are located in the adventitia, the external layer of the arterial wall. The study demonstrates an increase in axonal density and noradrenaline concentration within the adventitia, especially in the regions of ATLOs, in human atherosclerotic coronary arteries. This indicates that the dysregulated nervous system is key to the advancement of atherosclerosis by contributing to the development of ATLOS and their innervation (Nettersheim and Ley, 2022). ATLOs form extensive neuroimmune cardiovascular interfaces (NICIs) with the peripheral nervous system, while in mice, NICIs establish a structural arterial brain circuit (ABC). Certain elements of the peripheral nervous system get triggered during atherosclerosis process, indicating that their activity might be fostering the disease’s progression. Furthermore, the interruption of a segment of the sympathetic nervous system (SNS) through celiac ganglionectomy causes NICIs to disintegrate, reduces disease progression, and enhances plaque stability. These results indicate that therapeutic interventions targeting the ABC could mitigate the development of atherosclerosis (Mohanta et al., 2022). The SNS release neurotransmitters such as norepinephrine at their nerve endings, which increases vascular contractility and blood pressure, thereby promoting the development of atherosclerosis. Additionally, sympathetic nerve activity may further promote atherosclerosis development by enhancing inflammation and oxidative stress, which can disrupt vascular endothelial function and blood flow dynamics (Wang et al., 2023e). In ApoE^−/−^ mice that are given a HFD, the persistent activity of the SNS exacerbates oxidative stress in ECs by increasing blood levels of norepinephrine and the activity of mitochondrial monoamine oxidase A (MAO-A), culminating in mitochondrial dysfunction and inflammatory responses. Norepinephrine-activated MAO-A boosts the generation of ROS, particularly H_2_O_2_, which leads to DNA oxidative damage and the activation of NF-κB. Consequently, this process boosts the synthesis of molecules associated with inflammation in the ECs. Moreover, the activation of MAO-A also suppresses the expression of the mitochondrial function regulator PGC-1α, exacerbating mitochondrial dysfunction, thereby promoting the phenotypic changes of ECs towards atherosclerosis and the progression of atherosclerotic disease (Li et al., 2021).

Exercise markedly bolsters the nervous system, promoting balance within the autonomic nervous system, especially by augmenting parasympathetic nervous system activity and dampening SNS activity, which exerts beneficial effects on the cardiovascular system (Matei et al., 2022). Additionally, aerobic exercise has a positive impact on the autonomic nervous system in mice affected by atherosclerosis-induced autonomic impairment. The study shows that aerobic exercise training improves baroreflex sensitivity (BRS), an important marker of autonomic nervous system function that reflects the body’s ability to adapt to blood pressure changes. In this study, middle-aged ApoE^−/−^ mice without training showed lower baroreflex sensitivity in contrast to their younger counterparts, while trained middle-aged mice displayed baroreflex sensitivity similar to or better than that of young mice (Nascimento-Carvalho et al., 2024). In general, exercise may reduce atherosclerosis by modulating the nervous system (Figure 6).

### 2.9 Effect of exercise-modulated immune function on ameliorating atherosclerosis

Immune cells such as macrophages, T cell subsets, and B cells contribute to the immune microenvironment of atherosclerosis, encompassing both pro-inflammatory cells that drive disease progression and immunoregulatory cells that exert protective effects. Exercise may alleviate vascular inflammation and plaque formation by enhancing the activity of anti-inflammatory cells while suppressing pro-inflammatory cell functions. Additionally, exercise promotes efferocytosis to accelerate the clearance of apoptotic cells and mitigate inflammatory responses, thereby further inhibiting the progression of atherosclerosis.

#### 2.9.1 Immune response

In addition to inflammatory macrophages, various T cell subsets like CD4^+^ T helper 1 (Th1) cells and Tregs, play distinct roles in atherosclerosis. Th1 cells typically have a pro-atherosclerotic effect, while Tregs exhibit anti-atherosclerotic properties. Other immune cells, such as neutrophils, natural killer cells (NK cells), B cells, and dendritic cells (DCs) (Zhang X. et al., 2024), may also contribute to the pathogenesis of atherosclerosis (Tsukamoto et al., 2023). These immune cells collectively shape the complex immunological microenvironment of atherosclerosis (Fernandez and Giannarelli, 2022). Under conditions of hypercholesterolemia, neutrophils release neutrophil extracellular traps (NETs), promoting inflammation and endothelial damage, which may lead to plaque instability and exacerbate the development of atherosclerotic lesions. Tregs play a crucial defensive role in atherosclerosis by dampening inflammatory responses and fostering immune tolerance. Importantly, hypercholesterolemia may affect the phenotype and differentiation of T cells, encouraging the differentiation of Th1 and Th17 while reducing the number of Tregs. DCs are involved in antigen presentation, which stimulates Th1 and Th17 cells and inhibits Tregs (Aguilar-Ballester et al., 2020). In addition, natural IgM antibodies produced by B1 cells typically exhibit anti-atherosclerotic properties, whereas B2 cells (conventional B cells) may promote lesion development by producing cytokines such as TNFα or by supporting atherogenic T cells (Chen et al., 2020). In summary, the pathological progression of atherosclerosis is significantly influenced by immune cell function.

Exercise can modulate the immune response of immune cells. Physical activity is shown to increase the circulation of anti-inflammatory monocytes in the context of atherosclerosis, enhance the expression of IL-10 by Tregs, and alter the T cells balance by increasing the levels of circulating Tregs and inducing the polarization of anti-atherosclerotic Th2 cells, leading to the release of more immunosuppressive cytokines. Additionally, exercise curbs the pro-inflammatory monocytes, thereby diminishing vascular injury and the development of plaques (do Brito Valente et al., 2021). Overall, exercise may activate anti-atherosclerotic immune cells and inhibit pro-atherosclerotic immune cells, thus alleviating atherosclerosis (Figure 6).

#### 2.9.2 Efferocytosis

Efferocytosis involves phagocytes eliminating apoptotic cells, ensuring tissue homeostasis and resolving inflammation (Adkar and Leeper, 2024; Ngai et al., 2023). In the progression of atherosclerosis, disturbed flow patterns impair Mer tyrosine kinase (MerTK)-mediated efferocytosis in ECs. MerTK plays a pivotal role as both a receptor and a signaling protein critical for the removal of apoptotic cells. It is found that when blood flow exhibits disturbed or oscillatory shear stress, MerTK expression is suppressed in human aortic ECs (HAECs), affecting their ability to phagocytose apoptotic cells. This impaired efferocytosis can cause apoptotic cells to gather in the vessel wall, triggering secondary necrosis and pro-inflammatory responses, ultimately exacerbating atherosclerosis (Wu et al., 2024). These results suggest that enhanced efferocytosis through various pathways contributes to the amelioration of atherosclerosis.

Exercise can promote the efferocytosis process to facilitate the clearance of apoptotic cells, thereby mitigating inflammation. Extended periods of running could potentially halt the natural breakdown of triggering receptor expressed on myeloid cells 2 (Trem2) in the hippocampus of mice, reducing the amount of Trem2 released from the cell surface into the plasma. This implies that exercise may maintain the levels of Trem2 protein on the cell surface (Zhang et al., 2022). Additionally, Trem2 serves as a transmembrane molecule found in hepatic macrophages originating from both bone marrow and embryonic sources. Myeloid Trem2 in pro-inflammatory macrophages activates ras-related C3 botulinum toxin substrate 1 (Rac1) through the cyclooxygenase 2/prostaglandin E2 (COX2/PGE2) pathway, promoting the phagocytosis of apoptotic cells accumulated following hepatic ischemia-reperfusion injury in mice, and facilitating macrophage efferocytosis to suppress inflammation (Han et al., 2023). Changes in developmental endothelial locus-1 (DEL-1) expression levels in human skeletal muscle tissue before and after exercise, as observed through microarray analysis and RNA sequencing, have revealed that DEL-1 is a myokine secreted by muscles during physical activity, with its expression increasing post-exercise in skeletal muscle. In a mouse model of thoracic aortic dissection (TAD), the deficiency of DEL-1 impairs the ability of macrophages to clear apoptotic VSMCs. DEL-1 facilitates the recognition and phagocytosis of apoptotic VSMCs by macrophages through its interaction with phosphatidylserine (PtdSer) on the surface of apoptotic VSMCs and by binding to the ITGAV-ITGB3 integrin on macrophages. Consequently, DEL-1 produced by exercise may promote efferocytosis by macrophages following aortic injure (Kwon et al., 2024; Yin et al., 2024). Overall, exercise may have potential effects in alleviating atherosclerosis through the promotion of efferocytosis (Figure 6).

### 2.10 Other mechanisms of exercise-mediated alleviation of atherosclerosis

Exercise may alleviate atherosclerosis through multiple other molecular mechanisms, including the regulation of m6A RNA modifications and the upregulation of peroxisome proliferator-activated receptors (PPARs) and PGC-1α expression and activity, thereby suppressing inflammation and oxidative stress. Additionally, exercise may activate autophagy, facilitating the clearance of damaged cells and contributing to vascular health maintenance.

#### 2.10.1 N6-methyladenosine (m6A)

m6A, a common internal modification found in mRNA and non-coding RNAs in mammals, is crucial for modulating various biological processes (Zhang YS. et al., 2024). It is found that m6A-related enzymes such as methyltransferase-like 14 (METTL14) were upregulated in the aortic intima of patients with atherosclerosis (Xu et al., 2024). METTL14, as another member of the m6A methyltransferase family, can exacerbate the development of atherosclerosis by promoting inflammation in ECs. The study demonstrates that in the aortas of atherosclerotic mice, circular RNA circMETTL14 (11)S plays a crucial role in upregulating METTL14 expression and activates METTL14 transcription by binding to the transcription factor SOX2, thus enhancing METTL14-driven m6A methylation and stability of C-X-C chemokine receptor type 4 (CXCR4) mRNA. This m6A methylation modification enhances CXCR4 mRNA stability, thus promoting the inflammatory response in ECs (Kang and Dong, 2024). These results suggest that m6A methyltransferases promote the translation of various RNAs, enhancing the expression of specific proteins and activating different downstream pathways that promote inflammation, thereby exacerbating atherosclerosis.

Exercise can mitigate atherosclerosis by suppressing m6A levels. Exercise has been shown to diminish the activity and expression of METTL14 in the thoracic aorta of atherosclerotic mice, thereby inhibiting METTL14-mediated m6A modification on NEAT1 mRNA, which suppresses the expression of NEAT1, and consequently inhibits pyroptosis in ECs to alleviate atherosclerosis (Yang et al., 2023). In summary, exercise may regulate the m6A levels on the mRNAs of some specific genes by affecting the expression level of m6A methyltransferases, which may contribute to the amelioration of atherosclerosis. Further investigation is warranted into how exercise affects the expression of the m6A-related enzymes and effectors to control the pathogenesis of atherosclerosis (Figure 6).

#### 2.10.2 PPARs

The three subtypes of PPARs—PPAR-α, PPAR-β/δ, and PPAR-γ—hold potential as therapeutic targets for combating atherosclerosis (Guo et al., 2022). PPAR-α agonists are shown to reduce atherosclerotic lesions and hepatic fat accumulation in the ApoE^−/−^FXR^−/−^ mouse model by promoting β-oxidation of fatty acids and increasing liver to absorb fatty acids from plasma. This effect is linked to a rise in the levels of hepatic fatty acid transport proteins CD36 and fatty acid transport protein 1 (FATP1), along with the downregulation of apolipoproteins ApoC2 and ApoC3 in the liver. The above processes collaboratively enhanced the breakdown of fatty acids and reducing the accumulation of TG (Lee et al., 2021).

Exercise can upregulate the expression or activity of PPARs. A study is conducted to assess the expression of PPARs mRNA in the aortic tissue of ApoE^−/−^ mice following 3 months of swimming training. The findings reveal that swimming training significantly upregulated the expression of vascular PPAR-γ, which exhibited a negative correlation with the area of atherosclerotic plaques (Szostak et al., 2016). Additionally, low-intensity exercise by 17 sedentary individuals promoted the polarization of M2 macrophages through the PPAR-γ/PGC-1 coactivator-mediated signaling pathway. M2 macrophages are located in more stable positions within atherosclerotic plaques, indicating that exercise-related PPAR-mediated signaling effects can be considered beneficial in the context of atherosclerosis (Yakeu et al., 2010). Aerobic exercise has been shown to effectively mitigate inflammation in mice fed a HFD by activating the signaling pathway and enhancing PPAR-γ activity (Diniz et al., 2021). In general, exercise has the potential to promote PPARs expression or activity, thereby improving lipid metabolism and slowing the advancement of atherosclerosis.

#### 2.10.3 PGC-1α

PGC-1α plays an essential role as a coactivator in regulating mitochondrial biogenesis and energy metabolism. In VSMCs, it has demonstrated remarkable efficacy in stifling the production of ROS, curbing cellular proliferation and migration, and suppressing the inflammatory response induced by ox-LDL (Gan et al., 2022; Zhang X. et al., 2021). Exercise can significantly enhance the upregulation of PGC-1α expression (de Smalen et al., 2023). Different types of exercise training, including aerobic exercise, resistance training, whole-body vibration (WBV), and electrical stimulation (ES), have the capability to trigger the Sestrin2 (Sesn2)/AMPK/PGC-1α signaling cascade within the prefrontal cortex of mice, with resistance exercise showing the most significant effect. Resistance exercise may upregulate SESN2 expression, thereby activating the AMPK/PGC-1α signaling pathway to improve mitochondrial function and neuroprotection (Feng et al., 2023). A study shows that a 10-week exercise training triggers a series of complex biological effects in mice skeletal muscle cells through increased expression of PGC-1α. These effects include enhanced lipolysis and fatty acid oxidation, increased mitochondrial biogenesis, cellular signaling, and antioxidant responses (Mozaffaritabar et al., 2024). Therefore, PGC-1α-induced beneficial effects may play a role in the exercise-induced improvement of atherosclerosis.

#### 2.10.4 Autophagy

Autophagy is a cellular self-digestive mechanism that breaks down and recycles damaged or aged organelles and proteins within cells by forming autophagosomes. This process is key to sustaining cellular homeostasis. During the pathological progression of atherosclerosis, autophagy plays a pivotal role in safeguarding cells against oxidative damage, curbing apoptosis, and bolstering by eliminating impaired organelles and mitigating inflammatory reactions. Nevertheless, defects in autophagy can result in decreased clearance of misfolded proteins and damaged organelles, triggering inflammatory responses and cell death, thus promoting the development of atherosclerosis (Zhu et al., 2024).

Exercise can activate autophagy-related pathways, promoting autophagy and alleviating atherosclerosis. Studies have revealed that 16 weeks of voluntary exercise reduced serum levels of IL-1 receptor antagonist (IL-1RA) and elevates IL-1α levels in HFD-fed ApoE^−/−^ mice, thereby enhancing autophagy in ECs. Upon exposure to serum derived from exercise-trained ApoE^−/−^ mice or recombinant IL-1α and IL-1β proteins. This includes an upswing in LC3 mRNA transcription as well as elevated levels of LC3-I and LC3-II proteins, indicating enhanced autophagic activity in the HUVECs, thereby alleviating atherosclerosis (Okutsu et al., 2021). Additionally, after long-term swimming training, there is a marked upsurge in the levels of autophagy-related markers, LC3 and Beclin-1, detectable at both the mRNA and protein levels within the aorta. This surge corresponded with a boost in autophagic activity in ECs and effectively stifled the development of atherosclerotic plaques in the aortas of ApoE^−/−^ mice (Li et al., 2020b). In summary, exercise can upregulate autophagy markers like LC3 and Beclin-1, thus boosting autophagy and mitigating atherosclerosis.

### 2.11 Interaction of exercise and diet in the alleviation of atherosclerosis

Exercise and diet are key strategies for the prevention and management of atherosclerosis. Emerging evidence suggests that combining these two interventions may exert synergistic effects, providing greater benefits for vascular function. Through the coordinated regulation of lipid metabolism, endothelial function, and inflammatory responses, regular physical activity together with an appropriate dietary pattern can more effectively slow the progression of atherosclerosis.

One study involves patients with metabolic syndrome (MetS), an important risk factor for cardiovascular diseases, who are randomly assigned to two groups: one group receives a 12-week low-calorie Mediterranean diet (MeD), while the other group receives the same diet combined with moderate-to-high-intensity endurance training (MeDE). The results show that after 12 weeks, the number of EPCs significantly increased in the MeDE group, with a greater increase compared to the MeD group. Moreover, the reduction in TG is more pronounced in the MeDE group. Notably, ischemic reactive hyperemia (IRH), which is used to assess microvascular endothelial reactivity, improved significantly in the MeDE group, indicating that the combination of diet and exercise interventions can enhance vascular endothelial function, thereby improving atherosclerosis (Fernández et al., 2012). Another study involves obese adolescents aged from 12 to 18 years, divided into an intervention group and a standard care group. The intervention group receives a 10-month regimen of dietary restriction (1,500–1,800 kcal/d) and exercise training, followed by assessments of their vascular endothelial function and changes in circulating endothelial progenitor cells (EPCs) and endothelial microparticles (EMPs). EPCs can replace lost ECs, thereby alleviating endothelial dysfunction. EMPs are released discharged into the bloodstream from active or apoptotic ECs in the obese population. The increased number of EMPs reflects the damage or activation state of ECs, thereby indicating endothelial dysfunction. The results indicates that EPCs significantly increase in the initial phase of the intervention, reflecting the release of more EPCs from the bone marrow to participate in vascular repair. As treatment continued, there is a significant decrease in the number of EMPs, indicating reduced endothelial damage and improved vascular repair processes (Bruyndonckx et al., 2015). The evidence above suggests that diet-plus-exercise can promote vascular repair and improve blood lipids, thereby reducing the risk of atherosclerosis.

Moreover, intermittent fasting (IF), as a metabolic intervention strategy, has been extensively studied and has been shown to significantly improve body weight, lipid profiles, and blood pressure, thereby reducing the risk of CVDs (Kazeminasab et al., 2024). In the context of atherosclerosis, IF can reduce LDL-C and attenuate systemic inflammation, such as the suppression of IL-6 and TNFα, which may exert anti-atherogenic effects (Moro et al., 2021). However, it is shown that IF, although effective in preventing diet-induced obesity, fails to significantly reduce atherosclerotic plaque burden in the aortas of ApoE^−/−^ mice, indicating a potentially limited role of IF alone in atherosclerosis prevention (Inoue et al., 2021). Recent systematic reviews and meta-analyses of human studies consistently demonstrate that combining IF with exercise yields more pronounced improvements in cardiovascular and metabolic parameters such as body weight, fat mass, waist circumference, systolic blood pressure, and LDL-C, compared to either intervention alone. Furthermore, this combination enhances cardiorespiratory fitness, as reflected by increased maximal oxygen consumption (VO_2_ max), suggesting a synergistic effect (Khalafi et al., 2024). Therefore, IF combined with exercise may represent a more promising approach than single-modality interventions for the prevention of cardiovascular diseases, including atherosclerosis.

To achieve optimal intervention outcomes, in addition to developing personalized exercise programs based on individual needs, it is essential to implement targeted dietary interventions concurrently. Dietary strategies that limit energy intake, such as balanced nutrition and IF, play a critical role in cardiovascular protection. These approaches help reduce inflammation, improve lipid profiles and insulin sensitivity, and support vascular health. When combined with exercise, they may more effectively lower the risk of atherosclerosis and better alleviate existing atherosclerotic conditions, thereby preserving cardiovascular health.

### 2.12 Involvement of collateral vessel formation in exercise-mediated alleviation of atherosclerosis

The ameliorative effect of exercise training on atherosclerosis is manifested not only through the regulation of traditional factors but also via the promotion of collateral vessel formation, which compensates for blood flow in ischemic tissues.

A study has shown that moderate-intensity walking training upregulates circulating miR-142-5p, which promotes collateral formation by inhibiting transforming growth factor (TGF)-beta 2 (TGF-β2) expression, thereby reducing macrophage apoptosis, ameliorating the vascular wall microenvironment, and facilitating angiogenesis and endothelial function (Sieland et al., 2022). In patients with coronary artery disease, both high-intensity and moderate-intensity exercise training for 4 weeks significantly increased the coronary collateral flow index, enhanced exercise capacity, and raised the ischemic threshold, with comparable effects between the two training intensities. This suggests that regular exercise can alleviate myocardial ischemia by improving collateral circulation (Möbius-Winkler et al., 2016). Additionally, a study using ApoE-deficient mouse model confirmed that voluntary wheel running accelerated perfusion recovery after femoral artery ligation and increased macrophage accumulation around collateral vessels, indicating that exercise may promote collateral remodeling through inflammatory modulation (Bresler et al., 2019). These findings collectively demonstrate that exercise training facilitates collateral vessel formation through multiple mechanisms, including miRNA expression regulation and local inflammatory response modulation. This provides one more scientific basis for exercise intervention in atherosclerosis.

## 3 Discussion and conclusion

Currently, statins are the primary pharmacological treatment for atherosclerosis, but it is worth noting that they are also commonly associated with statin-associated muscle symptoms (SAMS) (Ferri and Corsini, 2025). The combination of exercise and statin therapy demonstrates synergistic effects in cardiovascular disease management. It is reported that moderate-intensity exercise not only does not exacerbate SAMS in statin users but may also improve muscle performance, mitochondrial function, and quality of life (Rosenson, 2023). Although drugs like atorvastatin may reduce mitochondrial biogenesis and ATP production in muscles, a 12-week combined moderate-intensity endurance and resistance training program can enhance citrate synthase activity, promote capillary density, and increase the proportion of type I muscle fibers in statin users, thereby counteracting these adverse effects (Macchi et al., 2022b; Rosenson et al., 2021). Therefore, it is suggested statin users maintain regular exercise, particularly for patients reporting muscle symptoms, as physical activity may serve as a key strategy to optimize treatment adherence and therapeutic outcomes. Critically, immediate cessation of the exercise activity is imperative upon manifestation of muscular discomfort during exercise, with prompt medical consultation strongly advised.​

Interestingly, various forms of physical activity yield distinct effects on the alleviation of atherosclerosis. A meta-analysis involving 2,420 participants assesses the effects of aerobic exercise, resistance exercise, combined aerobic and resistance exercise (CARE), and HIIT on patients with early-stage carotid atherosclerosis by analyzing CIMT and serum lipid-related indicators (Gao et al., 2023). Exercise programs notably lower TC and LDL-C levels, raise HDL-C levels and improve atherosclerosis. Subgroup analyses show that aerobic exercise reduces LDL-C and raises HDL-C, HIIT reduces LDL-C, and CARE increases HDL-C. Another study confirms that exercise decreases CIMT, underscoring the effectiveness of aerobic exercise and HIIT (Byrkjeland et al., 2016b). A systematic review of 26 studies (1,370 participants) finds that individuals exercising for more than 6 months experienced greater CIMT reductions (Wang et al., 2022b). Furthermore, the analysis encompasses six different types of exercise, including aerobic exercise and resistance training, CARE, HIIT, MICT, and endurance exercise, with aerobic exercise showing the most significant impact on reducing CIMT. Additionally, varying levels of exercise intensity are evaluated, with moderate intensity demonstrating the most favorable outcomes. Thus, this study provides evidence supporting the role of long-term moderate-intensity aerobic exercise as the most effective approach for reducing CIMT levels. Another study developed a model of early-stage atherosclerosis using ApoE-deficient mice fed with HFD, characterized by significant dyslipidemia. By comparing the effects of HIIT and MICT in atherosclerotic mice, both exercise modalities were found to significantly reduce TC and TG, improve dyslipidemia, and decrease ROS production and protein carbonylation. Among these, MICT exhibited unique advantages in increasing HDL-C (102). Therefore, the mitigation effects of exercise on atherosclerosis may depend on the type of exercise, with long-term, moderate-intensity aerobic exercise potentially offering greater benefits.​​

Notably, one study explored how aerobic exercise mitigates atherosclerosis in normal diet-fed APOE-deficient mice at different disease stages, revealing differential effects. In an early-stage atherosclerosis model (12-week-old mice receiving 10 weeks of exercise intervention), exercise significantly improved the lipid profile: reducing plasma TC by 16%, free cholesterol by 13%, TG by 35%, and phospholipids by 27%. More importantly, exercise reduced aortic sinus plaque stenosis and altered plaque composition. Collagen content increased 11-fold and elastin increased 3-fold, indicating enhanced plaque stability. In a late-stage atherosclerosis model (40-week-old mice receiving the same intervention), exercise still reduced plaque stenosis and volume, though to a lesser extent. However, exercise led to no significant changes in collagen, elastin, or VSMC content within the late-stage plaques. Regarding lipids, only free cholesterol and phospholipids showed mild reductions, with no significant differences observed in TC or TG. Similar to the early-stage model, tissue inhibitor of metalloproteinases-1 (TIMP-1) levels were also reduced, while TIMP-2 and inflammatory markers (macrophages, MCP-1) showed no significant changes. These findings demonstrate a time-dependent effect of exercise intervention. Early intervention not only improves dyslipidemia more dramatically but also promotes a stabilizing transformation in plaque structure. The results indicate that exercise exerts more pronounced mitigation effects on early-stage atherosclerosis. However, even in late-stage lesions where plaques maintain morphological rigidity, exercise can still reduce their volume, underscoring its irreplaceable clinical value even for advanced disease (Stanton et al., 2022).

A study investigating the effects of long-term, high-intensity endurance exercise on coronary artery health revealed a profound scientific controversy. Conventional understanding posits that endurance exercise significantly reduces cardiovascular risk by optimizing blood pressure, lipid profiles, and insulin sensitivity. However, empirical data indicate that athletes engaged in lifelong endurance training paradoxically exhibit a higher burden of coronary plaques.​​ The core of this contradiction lies in the observation that despite demonstrating exceptional cardiorespiratory fitness levels, lifelong athletes display greater plaque prevalence on coronary angiography. This particularly involves proximal non-calcified plaques and mixed plaques, types associated with elevated cardiovascular event risk (De Bosscher et al., 2023). This paradox underscores the complexity of exercise-induced effects on cardiovascular system, suggesting the importance of maintaining appropriate amount of exercise.

To sum up, exercise is a cost-effective, beneficial intervention that modulates metabolic and inflammatory pathways, providing numerous protective benefits (Meyer-Lindemann et al., 2023b). Engaging in structured exercise or moderate physical activity can assist in the prevention and management of atherosclerosis across different populations. Notably, this review synthesizes the molecular mechanisms through which exercise mitigates atherosclerosis, encompassing the regulation of exercise-induced factor expression, promotion of iWAT browning and BAT activation, elevation of blood lactate concentration to facilitate lactylation modification, and suppression of diverse cell death pathways. However, critical knowledge gaps persist, such as the unverified mechanisms by which exercise may attenuate atherosclerosis via elevated Lac-Phe levels, ferroptosis inhibition, modulation of miRNAs expression levels, the potential crosstalk and interplay between different exercise-induced pathways or effectors, and so on, necessitating experimental validation. Besides, few lines of evidence have been provided regarding the differentiation of the roles of exercise in atherosclerosis based on age and race. Moreover, future research should focus on developing precise, personalized exercise protocols. These protocols need dynamic adjustment based on disease severity, plaque stability, and individual functional reserves. Achieving this requires not only establishing scientific individual assessment systems but also relying on a deeper mechanistic understanding of how exercise ameliorates atherosclerosis at the molecular level. This will enable the effective translation of theoretical insights into clinical practice. In clinical practice, it is essential to conduct a comprehensive assessment of each patient’s health condition, physical fitness level, severity of atherosclerosis, and personal preferences. Based on this information, personalized diet and exercise plans should be developed to ensure that the recommended interventions are tailored to the specific circumstances of atherosclerosis patients. Regular monitoring of patient’s disease progression and adjustment of diet and exercise regimens based on intervention effects are crucial. Moreover, elucidating the underlying molecular mechanisms by which exercise exerts positive effects on atherosclerosis will facilitate the development of safer and more effective treatment and prevention protocols in the future. Clinicians are advised to stay informed about the latest research in this field, so as to timely update their knowledge base and apply new findings in practice.

## Glossary

AE: Aerobic exercise
α-KG: Alpha-ketoglutarate
ATLOs: Arterial tertiary lymphoid organs
AT1R: Ang II type 1 receptor
BAT: Brown adipose tissue
BMP4: Bone morphogenetic protein 4
CARE: Combined aerobic and resistance exercise
CIMT: Carotid intima-media thickness
DEL-1: Developmental endothelial locus-1
EC: Endothelial cell
EMPs: Endothelial microparticles
eNOS: Endothelial nitric oxide synthase
EPCs: Endothelial progenitor cells
ER: Endoplasmic reticulum
FGF21: Fibroblast growth factor 21
GLP-1: Glucagon-like peptide-1
GLP-1R: GLP-1 receptor
GLP-1RAs: GLP-1R agonists
GSDMD: Gasdermin D
GSH: Glutathione
HDL: High-density lipoprotein
HFD: High-fat diet
HIIT: High-intensity interval training
HUVECs: Human umbilical vein endothelial cells
ICAM-1: Intercellular adhesion molecule-1
IL-1β: Interleukin-1β
IL-6: Interleukin-6
LDL: Low-density lipoprotein
LPS: Lipopolysaccharide
MICT: Moderate-intensity continuous training
miRNAs: MicroRNAs
MMPs: Matrix metalloproteinases
m6A: N6-methyladenosine
NEAT1: Nuclear paraspeckle assembly transcript 1
Nrf2: Nuclear factor erythroid 2-related factor 2
ox-LDL: Oxidized low-density lipoprotein
PCSK9: Proprotein convertase subtilisin/kexin type 9
PGC-1α: Peroxisome proliferator-activated receptor γ coactivator-1α
PPARs: Peroxisome proliferator-activated receptors
PVAT: Perivascular adipose tissue
RAS: Renin-angiotensin system
sACE2: Soluble ACE2
SMC: Smooth muscle cell
SNS: Sympathetic nervous system
TC: Total cholesterol
TG: Triglycerides
Th1: T helper 1
TNFα: Tumor necrosis factor α
Tregs: Regulatory T cells
UCP1: Uncoupling protein 1
VCAM-1: Vascular cell adhesion molecule-1
WAT: White adipose tissue
WT: Wild type
